# A bis-terpyridine-based trimetallic Co/Fe/Ni metallo-supramolecular polymer for electrochemical detection of 4-nitrophenol: kinetic and mechanistic insights

**DOI:** 10.1039/d6ra08328e

**Published:** 2026-10-15

**Authors:** Najia Tahsin Kumkum, Surekha Chaudhary, Md. Monjorul Islam, Sheikh Jakia Nur Oishee, Abdul Awal, Md. Delwar Hossain, Bong-Joong Kim, A. J. Saleh Ahammad

**Affiliations:** a Department of Chemistry, Jagannath University Dhaka 1100 Bangladesh delwar@chem.jnu.ac.bd ajsahammad@chem.jnu.ac.bd; b Department of Materials Science and Engineering, Gwangju Institute of Science and Technology (GIST) Gwangju 61005 Korea kimbj@gist.ac.kr

## Abstract

A bis-terpyridine-based trimetallic Co/Fe/Ni metallo-supramolecular polymer (polyCoFeNi) was synthesized by one-pot coordination of Co, Fe, and Ni precursors with 4′,4‴-(1,4-phenylene)bis(2,2′:6′,2″-terpyridine) and investigated as an electrode modifier for electrochemical determination of 4-nitrophenol (4-NP). UV-vis spectroscopy, FTIR, FESEM, EDS, XPS, and viscosity measurements supported formation of a Co/Fe/Ni-containing coordination material with a rough, sponge-like morphology and metal–nitrogen coordination environments. Because the one-pot synthesis and available spectroscopic data do not establish an ordered Co–Fe–Ni sequence or unique mixed-valence states, the structural interpretation is kept conservative. The polyCoFeNi-modified glassy carbon electrode showed higher O1/*R*_2_ responses than the bare electrode and the investigated monometallic analogues. Cyclic voltammetry revealed an initial irreversible reduction followed by a reversible oxidation/reduction couple, while scan-rate analysis indicated mixed diffusion–adsorption control. Under optimized conditions, a linear response was obtained over 2–2000 µM with limits of detection of 0.28 and 0.65 µM for the anodic and cathodic processes, respectively. The modified electrode also showed good reproducibility, operational stability, selectivity, and satisfactory performance in spiked water samples. These results demonstrate the potential of Co/Fe/Ni-containing bis-terpyridine metallo-supramolecular materials as electrochemical interfaces for 4-NP sensing.

## Abstract 2

A bis-terpyridine-based Co/Fe/Ni metallo-supramolecular polymer enables sensitive electrochemical detection of 4-nitrophenol. Kinetic studies reveal a mixed diffusion–adsorption-controlled process, highlighting its potential for 4-NP sensing.

## Graphical abstract

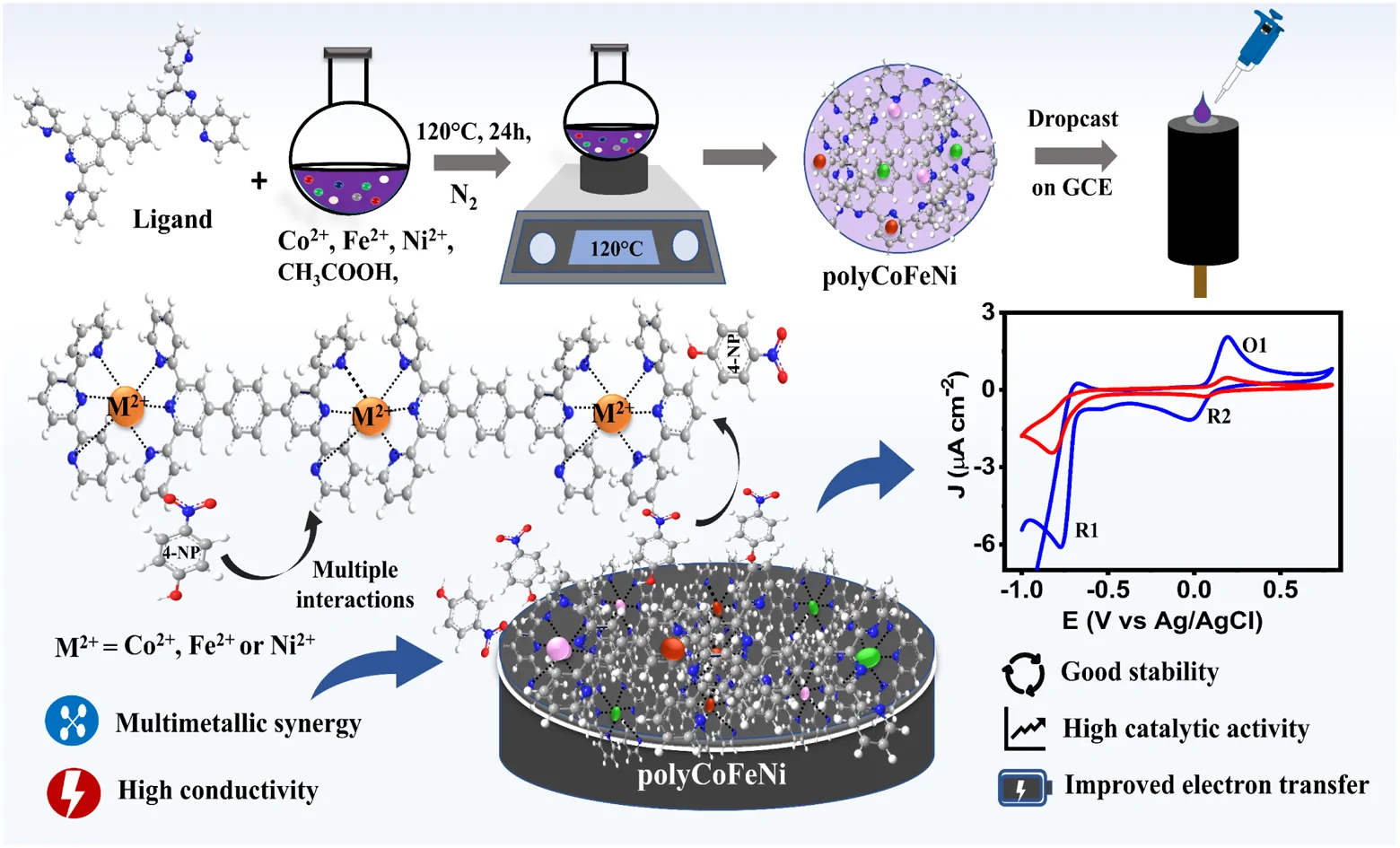

## 1. Introduction

The rapid expansion of industrialization and anthropogenic activities has substantially increased the discharge of untreated pollutants into the environment, posing serious threats to ecosystems and human health.^1,2^ The continuous release of industrial effluents disrupts aquatic biodiversity and ecological balance, while contaminating agricultural soils through runoff, ultimately reducing soil fertility and crop productivity.^3,4^ Among these pollutants, 4-nitrophenol (4-NP) recognized as one of the most toxic, persistent, and widely distributed nitroaromatic contaminants.^5^ Owing to its extensive use as an intermediate in the manufacture of dyes, pharmaceuticals, pesticides, and explosives, 4-NP is frequently detected in environmental matrices and can readily accumulate in the food chain.^6,7^

Exposure to 4-NP has been associated with severe health hazards, including methemoglobinemia (blue baby syndrome), liver and kidney dysfunction, skin irritation, carcinogenicity, and, in extreme cases, death.^8^ Consequently, both the World Health Organization (WHO) and the United States Environmental Protection Agency (USEPA) have identified 4-NP as a hazardous environmental pollutant requiring continuous monitoring and effective remediation.^9,10^ These concerns highlight the need for rapid, sensitive, and cost-effective analytical methods capable of detecting trace levels of 4-NP in environmental samples.

Several analytical techniques including high-performance liquid chromatography (HPLC),^11^ gas chromatography (GC),^12^ mass spectrometry,^13^ UV-vis spectrophotometry^14^ and electrophoresis^15^ have been developed for the determination of 4-NP. Although these techniques provide excellent sensitivity and analytical accuracy, they generally require expensive instrumentation, labor-intensive sample preparation, skilled operators, and centralized laboratory facilities.^16^ In contrast, electrochemical sensing has emerged as an attractive alternative because of its high sensitivity, rapid response, low cost, simple operation, and suitability for on-site environmental monitoring.^17^ The electrochemical redox behavior of 4-NP involves proton-coupled multi-electron transfer processes, making its analytical performance highly dependent on the electrocatalytic properties of the electrode material.^13^ Accordingly, the rational design of functional coordination polymers with tunable electronic properties and abundant electroactive sites has become an attractive strategy for improving electrochemical sensing performance.

Metallo-supramolecular polymers (MSPs) have attracted considerable attention as functional electrochemical materials, because they integrate the redox activity of transition metal centers with the structural versatility of coordination-driven polymeric chain.^18^ Unlike conventional polymers, the dynamic coordination bonds in MSPs enable broad tuning of their composition, topology, and electronic properties through the judicious selection of metal ions and organic ligands. Their architecture and physicochemical properties can be precisely tuned through the choice of metal ions, ligand structure, coordination geometry, solvent system, and assembly conditions.^19^ Such structural flexibility has enabled their application in chemical sensing, energy storage, electrocatalysis, water splitting, and dye-sensitized solar cells.^20^

Among various ligands, 4′,4‴-(1,4-phenylene) bis (2,2′:6′,2′-terpyridine) is particularly attractive because its two terpyridine coordination sites provide six nitrogen donor atoms connected through a rigid π-conjugated phenylene spacer, facilitating the construction of mono-, bi-, and multimetallic supramolecular polymer networks.^21^ The strong metal–ligand coordination together with the extended π-conjugated backbone can support electronic communication between coordinated metal centers and provides a useful platform for developing multifunctional coordination polymers with tunable redox properties.

Recent developments in coordination-based functional materials further demonstrate the versatility of metal–ligand assembly for environmental sensing, pollutant removal, and catalytic applications. For example, polyoxometalate-based supramolecular materials and composites have been developed as electrochemical sensing platforms for environmentally and biologically relevant analytes,^22^ while metal–organic frameworks (MOFs) have enabled contaminant recognition and removal through tunable coordination environments and host–guest interactions.^23^ Related coordination materials have also enabled sensitive recognition and catalytic transformation of phenolic pollutants,^24^ whereas MOF-based luminescent sensors highlight host–guest interactions and electron/energy-transfer processes as important recognition mechanisms for nitro-containing analytes.^25^ Coordination-mode control can further regulate the accessibility of metal active sites and thereby influence catalytic performance in metal–organic complexes.^26^ More broadly, metal–phenolic networks demonstrate how metal–ligand coordination and accessible metal redox states can generate multifunctional materials with tunable physicochemical properties.^27^ Collectively, these studies highlight that rational control of metal centers, ligand environments, and multicomponent interactions provides an effective strategy for constructing functional materials with enhanced molecular recognition, charge-transfer, adsorption, and catalytic properties.

Transition metals such as Co, Fe, Ru, Cu, and Ni have been widely incorporated into metallo-supramolecular polymers owing to their multiple accessible oxidation states, favorable redox chemistry, and efficient electron-transfer characteristics.^28^ Recently, monometallic, bimetallic, and trimetallic SMPs, including polyFe,^29^ polyNi,^30^ polyCu,^31^ polyNiCo,^32^ polyCoRu,^33^ polyNiRu,^34^ polyCoFe,^35^ and polyCoFeRu,^36^ have demonstrated excellent electrochemical performance for nitrite and sulfite sensing. These studies indicate that incorporating multiple transition-metal centers within a common coordination polymer chain can modify the redox and electrochemical properties of metallo-supramolecular polymers. Nevertheless, the synthesis and electrochemical application of a bis-terpyridine-based trimetallic Co/Fe/Ni metallo-supramolecular polymer for the detection of 4-NP have not yet been reported. Considering the distinct redox characteristics of Co, Fe, and Ni together with the conjugated bis-terpyridine polymer chain, a Co/Fe/Ni-containing supramolecular material may provide multiple electroactive environments and favorable analyte-surface interactions for the proton-coupled redox transformation of 4-NP.

In this work, we report the rational design and synthesis of a bis-terpyridine-based trimetallic Co/Fe/Ni metallo-supramolecular polymer (polyCoFeNi) and investigate its application as an electrode modifier for the electrochemical determination of 4-NP. The polymer was comprehensively characterized by UV-vis spectroscopy, FTIR, FESEM, EDS, XPS, and viscosity measurements, while its electrochemical properties were evaluated by cyclic voltammetry (CV) and electrochemical impedance spectroscopy (EIS). The electrochemical response of the polyCoFeNi-modified electrode was compared with those of the bare GCE and the corresponding monometallic materials to evaluate the effect of incorporating multiple metal centers. Furthermore, the redox mechanism, electron-transfer kinetics, analytical performance, operational stability, reproducibility, selectivity, and applicability to real water samples were systematically investigated, demonstrating the potential of Co/Fe/Ni-containing bis-terpyridine supramolecular materials as electrode modifiers for electrochemical sensing.

## 2. Experimental

### 2.1. Materials

All chemicals were of analytical grade and used as received without further purification. Methanol (CH_3_OH), acetic acid (CH_3_COOH), acetonitrile (C_2_H_3_N), dimethyl sulfoxide (DMSO), acetone (CH_3_COCH_3_), and hexane (C_6_H_14_) were purchased from Sigma-Aldrich (USA). Deionized water (DI) was used throughout the study for solution preparation and cleaning of glassware and electrode surfaces. Phosphate buffer solution (PBS) served as the supporting electrolyte for all electrochemical measurements, and its pH was adjusted by the dropwise addition of 0.1 M HCl or 0.2 M NaOH, as required. Potassium chloride (KCl), potassium ferricyanide (K_3_[Fe(CN)_6_]), sodium nitrite (NaNO_2_), ferrous acetate [Fe(OAc)_2_], cobalt acetate [Co(OAc)_2_], nickel perchlorate [Ni(ClO_4_)_2_], and 4′,4‴-(1,4-phenylene)bis (2,2′:6′,2′-terpyridine) were obtained from Sigma Aldrich (USA), Kanto, Wako or TCL.

Safety note: Nickel(ii) perchlorate, Ni(ClO_4_)_2_, should be handled with appropriate precautions because perchlorate salts are strong oxidizing agents and may cause fire or explosion hazards.

### 2.2. Instrumentation

Electrochemical measurements were performed using a conventional three-electrode configuration, consisting of a bare GCE or polyCoFeNi-modified GCE (polyCoFeNi_GCE) as the working electrode, an Ag/AgCl (saturated KCl) electrode as the reference electrode, and a platinum wire as the counter electrode. All electrochemical experiments were carried out using a CHI 660E electrochemical workstation (CH Instruments, USA).

The surface morphology of the synthesized polymer was examined by FESEM (JSM-7600F, JEOL, Japan). FTIR spectra were recorded on an IRTracer-100 spectrophotometer (Shimadzu, Japan) using the KBr pellet method to identify the characteristic functional groups. UV-vis absorption spectra were collected using a UV-1800 double-beam spectrophotometer (Shimadzu, Japan). Ultrasonic dispersion was performed using a Sonic-360 ultrasonicator. XPS measurements were conducted using a K-Alpha XPS system (Thermo Fisher Scientific, USA) equipped with a monochromatic Al Kα X-ray source under ultrahigh-vacuum conditions.

### 2.3. Synthesis process of polyCoFeNi

PolyCoFeNi was synthesized by dissolving ferrous acetate [Fe(OAc)_2_] (3.7 mmol), cobalt acetate [Co(OAc)_2_]·4H_2_O (3.7 mmol), nickel perchlorate [Ni(ClO_4_)_2_]z·6H_2_O (3.7 mmol), and 4′,4‴-(1,4-phenylene)bis(2,2′:6′,2″-terpyridine) (11.1 mmol) in acetic acid. The reaction mixture was heated at 120 °C for 24 h under a nitrogen atmosphere with continuous stirring. After completion of the reaction, the product was cooled to room temperature, washed three times with 10 mL of *n*-hexane, and dried at room temperature to obtain the polyCoFeNi powder in 95.5% yield. The one-pot synthesis is illustrated schematically in Scheme 1.

**Scheme 1:**
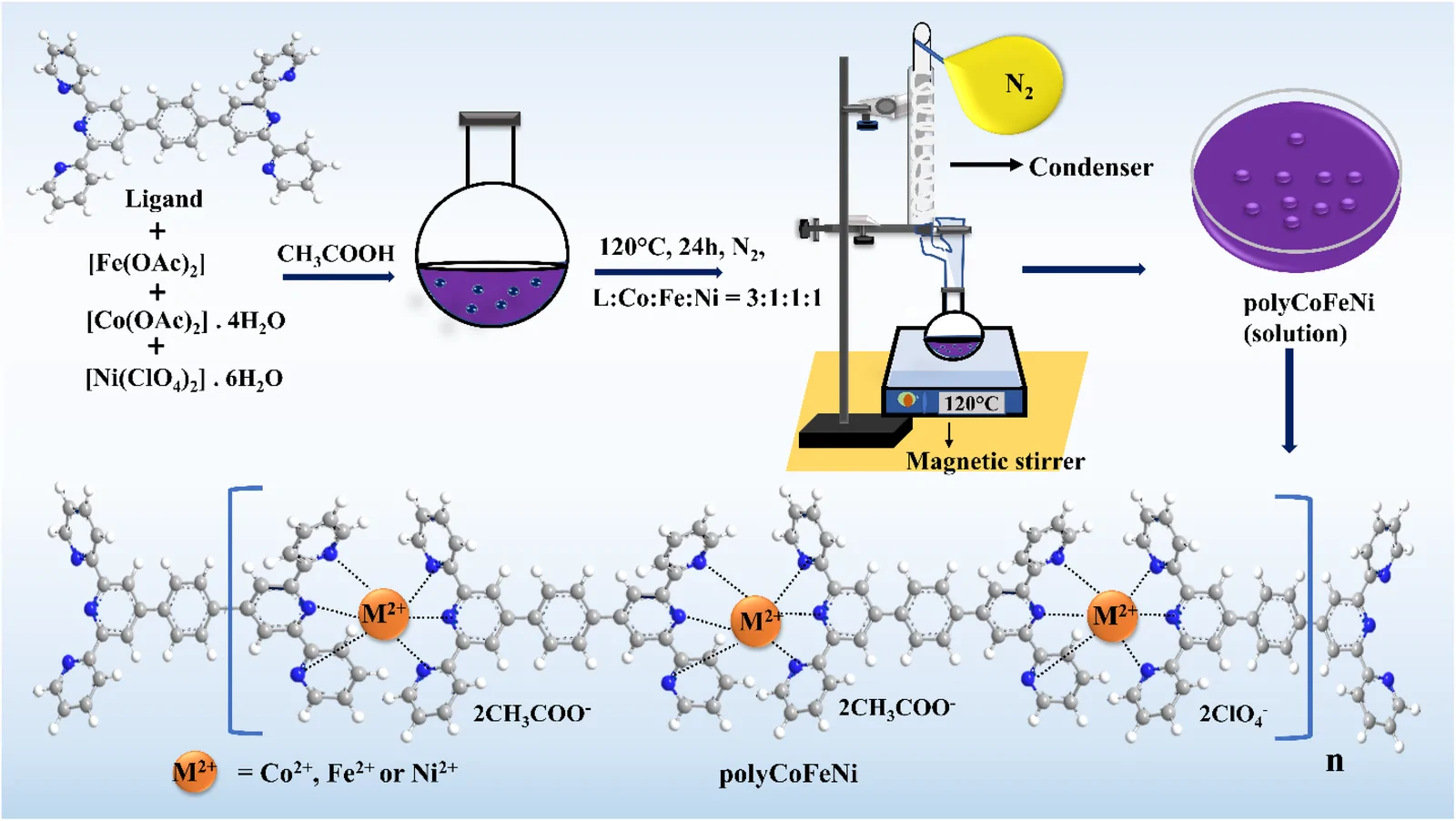
Schematic representation of the synthesis and coordination structure of the Co/Fe/Ni-containing bis-terpyridine metallo-supramolecular polymer (polyCoFeNi), where M represents Co, Fe, or Ni. The schematic illustrates metal–terpyridine coordination and does not imply a defined sequence of the individual metal ions along the polymer chain.

### 2.4. Fabrication of the polyCoFeNi-modified GCE

To prepare the polyCoFeNi suspension, 2 mg of polyCoFeNi was dispersed in a mixture of 750 µL methanol and 250 µL deionized (DI) water, followed by sonication at 30 °C for 40 min to obtain a homogeneous dispersion. Prior to modification, the glassy carbon electrode (GCE) was polished with a 0.05 µm alumina slurry on a polishing cloth for 10 min, thoroughly rinsed with DI water, and dried at room temperature. Subsequently, 5 µL of the polyCoFeNi suspension was drop-cast onto the cleaned GCE surface and allowed to dry at room temperature for 10 min. The resulting polyCoFeNi-modified GCE (polyCoFeNi_GCE) was then used for all subsequent electrochemical measurements (Scheme 2).

**Scheme 2:**
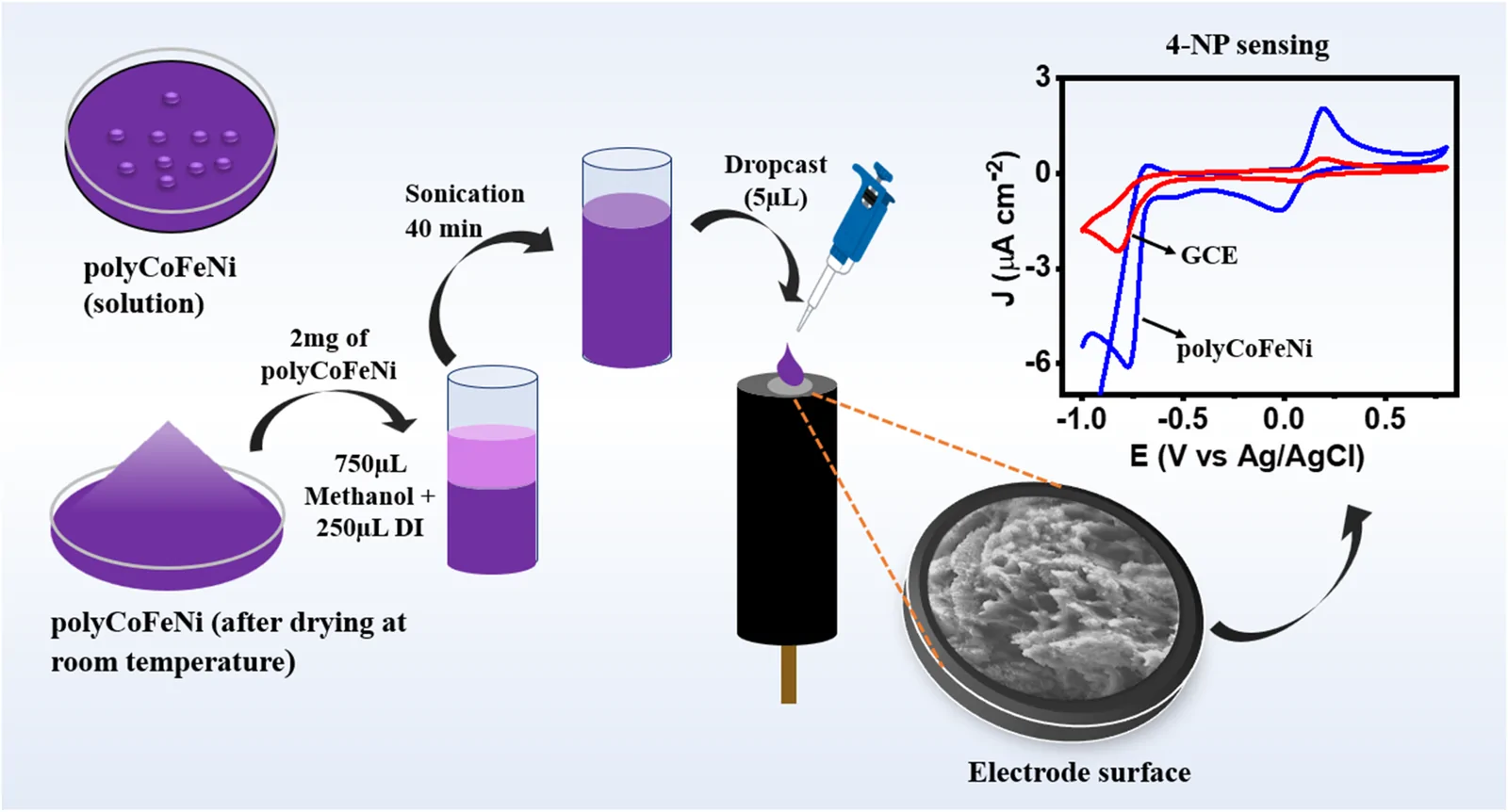
Schematic illustration of the fabrication of the polyCoFeNi-modified GCE.

## 3. Results and discussions

### 3.1. Characterization of the polyCoFeNi

The formation, composition, functional groups, and surface features of polyCoFeNi were systematically investigated using UV-vis spectroscopy, FTIR spectroscopy, viscosity measurements, FESEM, EDS, and XPS analyses. Together, these techniques support formation of a Co/Fe/Ni-containing bis-terpyridine supramolecular material and provide information on its morphology, elemental composition, and coordination environment, as discussed below.

The trimetallic polyCoFeNi was designed by integrating Co, Fe, and Ni ions into a bis-terpyridine coordination polymer chain to combine the complementary redox properties of the three transition metals within a single supramolecular polymer. The rigid π-conjugated bis-terpyridine ligand not only serves as a multidentate coordination unit but also promotes electronic communication between adjacent metal centers through the extended polymer backbone. Such a coordination architecture is expected to generate multiple electroactive sites, facilitate electron delocalization, and improve charge-transport characteristics compared with the corresponding monometallic polymers. Moreover, the coexistence of different transition-metal centers provides a favorable environment for synergistic redox interactions, which are anticipated to enhance the electrocatalytic activity toward 4-NP. Therefore, comprehensive structural characterization is essential to evaluate the formation and composition of the heterometallic coordination material and relate the observed structural features to its electrochemical behavior.

#### 3.1.1. UV-vis spectroscopic and FTIR analyses

The stepwise complexation of the bis-terpyridine ligand with Co(ii), Ni(ii) and Fe(ii) ions is shown in Fig. 1a. Upon the gradual addition of Co(ii) salt to the ligand solution, the absorption intensity increased around 285 nm, which can be assigned to metal-to-ligand charge transfer (MLCT), confirming the initial metal–ligand coordination. Subsequent addition of Ni(ii) salt to the Co-ligand system produced a new absorption band at 327 nm, indicating a change in the ligand electronic environment upon Ni(ii) addition.

**Fig. 1:**
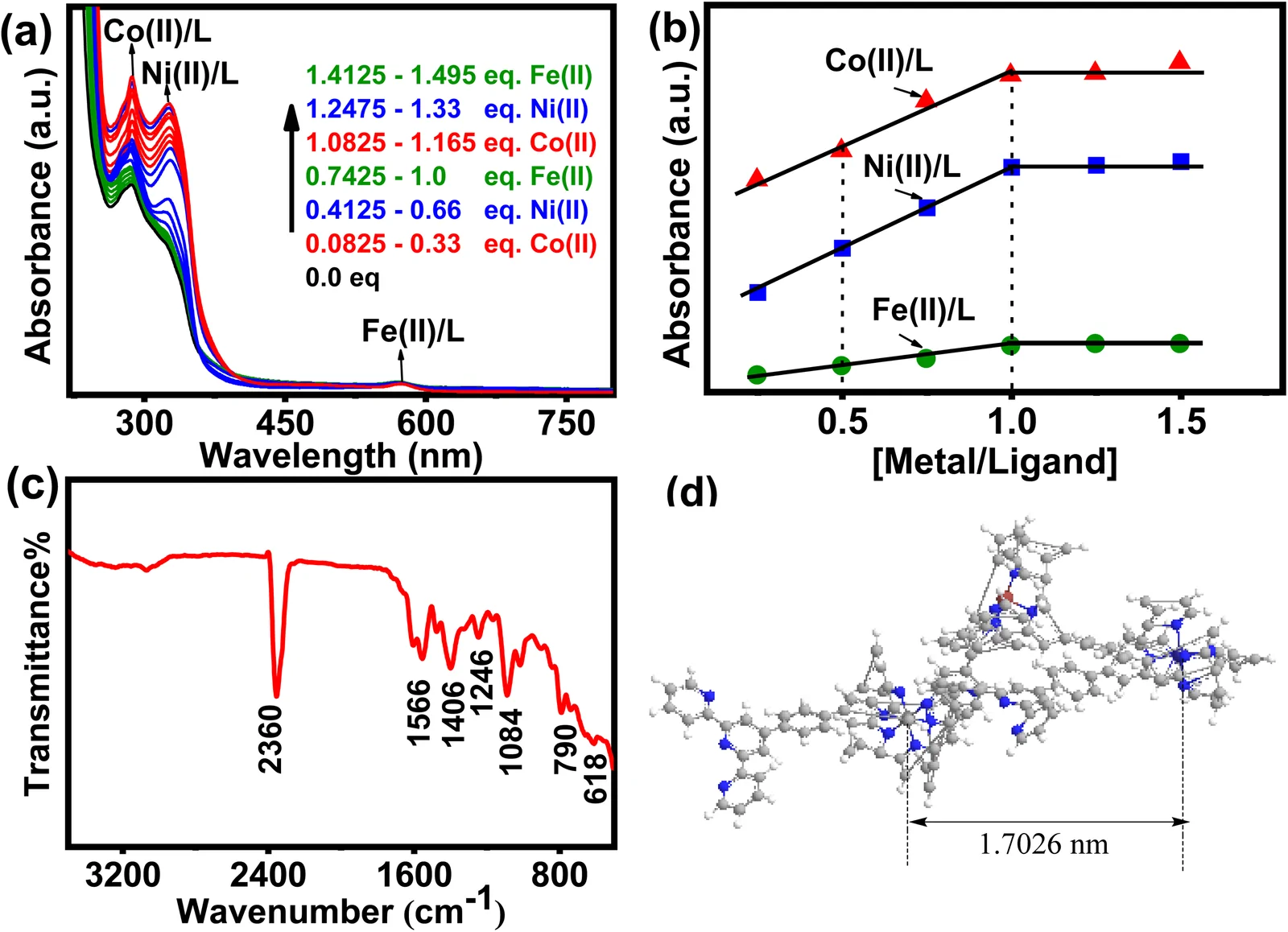
(a) Changes in spectrum obtained by addition of Co(ii) acetate, Ni(ii) perchlorate and Fe(ii) acetate, in the ligand solution during UV/vis titration. (b) Accumulation of Co, Ni and Fe ions to the ligand gives absorbance change at 285 nm, and 327 nm and 574.5 nm, respectively. (c) FT-IR spectra of the polyCoFeNi. (d) Average intermetallic distance through molecular modeling.

Finally, after the addition of Fe(ii) salt, a distinct absorption band appeared at 574.5 nm, indicating a further change in the coordination environment after Fe(ii) addition. The presence of clear isosbestic points during the UV-vis titration shows discrete spectral changes during the titration sequence. The observed absorption features are consistent with previously reported SMPs.^30,36^ In addition, as shown in Fig. 1b, the absorbance of the characteristic bands increased progressively with increasing metal/ligand ratio and reached saturation at approximately a 1 : 1 stoichiometric ratio, indicating metal–ligand interaction under the titration conditions. These titration experiments probe sequential binding behavior in solution and should not be interpreted as direct evidence for an ordered Co–Fe–Ni sequence in the isolated one-pot product.

FTIR analysis was performed to identify the functional groups and coordination-related bonding features of polyCoFeNi (Fig. 1c). The band at 2360 cm^−1^ is assigned to O=C=O stretching vibration, most likely arising from atmospheric CO_2_.^37^ The prominent bands in the 1600–1400 cm^−1^ region are attributed to C=N, C=C, and C–H stretching vibrations, consistent with the presence of terpyridine moieties in the coordination material.^38^ The bands at 1246 and 1084 cm^−1^ correspond to C–C and C–N stretching vibrations, respectively.^39^ The lower-wavenumber bands at 790 and 618 cm^−1^ are more conservatively assigned to ligand ring-deformation/coordination-related modes and are not used as standalone evidence for Fe–O bonding.^29,40^

The molecular weight of the polyCoFeNi was calculated using the following Mark–Houwink–Sakurada equation:^32^

1 [*η*] = *K*M^*a*^

where [*η*], *K*, and a represent the intrinsic viscosity, Mark–Houwink constant, and Mark–Houwink exponent, respectively. The values used were 5.21 dL g^−1^, 9.66 × 10^−5^ dm^3^ g^−1^, and 0.742, respectively. Based on these values, the molecular weight of polyCoFeNi was calculated to be 23.6 × 10^5^ Da, corresponding to approximately 979 repeating units and an average polymer chain length of 1032 nm. Molecular modeling further suggested an average intermetallic distance of 1.7026 nm (Fig. 1d).

#### 3.1.2. FESEM and EDS analysis

The surface morphology of polyCoFeNi was examined by FESEM at both low and high magnifications (Fig. 2a and b). The images reveal a three-dimensional sponge-like, wrinkled, and interconnected network structure, with pronounced surface roughness and interconnected micron-scale voids.^41,42^ The polymer chain consists of agglomerated, dendritic, and rough surface features with numerous interconnected voids. Because FESEM alone does not quantify internal porosity or specific surface area, these images are interpreted primarily as evidence of surface roughness and morphological heterogeneity.

**Fig. 2:**
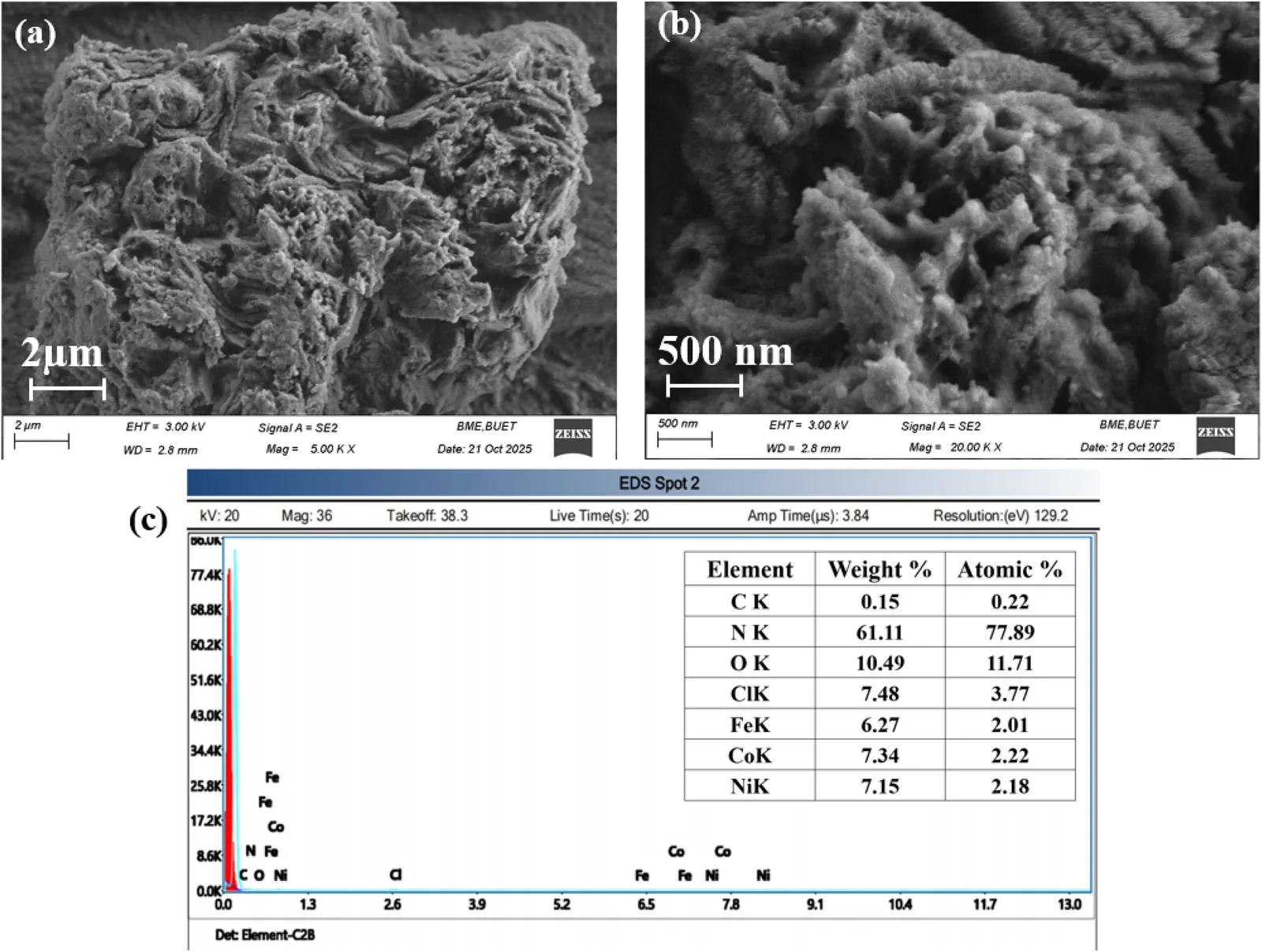
FESEM images at (a) low and (b) high amplifications, and (c) the atomic percentage of polyCoFeNi.

EDS analysis was performed to determine the elemental composition and relative elemental abundance of polyCoFeNi (Fig. 2c). The EDS spectrum shows signals attributable to C, N, O, Cl, Fe, Co, and Ni and confirms the simultaneous presence of the three transition metals in the analyzed region. Because light-element quantification by EDS is sensitive to background correction and peak assignment, the C/N atomic percentages are therefore not used here to establish stoichiometry or compositional purity. The detected chlorine may originate from the Ni(ClO_4_)_2_ precursor used during synthesis.

#### 3.1.3. XPS analysis

XPS was performed to investigate the elemental composition, chemical bonding, oxidation states, and coordination environment of polyCoFeNi. Since the polymer was deposited onto the electrode by a simple drop-casting process, the coordination polymer chain of the synthesized Co/Fe/Ni bis-terpyridine supramolecular polymer was expected to remain unchanged. XPS therefore provides direct evidence of the chemical states of the coordinated metal centers, which are closely related to the electrochemical behavior of the material.

The survey spectrum (Fig. 3a) confirms the presence of Co, Fe, Ni, N, C, and O, in good agreement with the EDS results, thereby verifying the successful formation of the heterometallic supramolecular polymer. The high-resolution Co 2p spectrum (Fig. 3b) exhibits the characteristic Co 2p_3/2_ and Co 2p_1/2_ doublet. The peaks centered at 780.2 and 794.3 eV are assigned to Co^3+^, whereas those at 783.7 and 797.1 eV correspond to Co^2+^.^43^ The satellite peaks located at 786.7 and 801.7 eV further confirm the presence of cobalt species within the coordination polymer chain.^44^

**Fig. 3:**
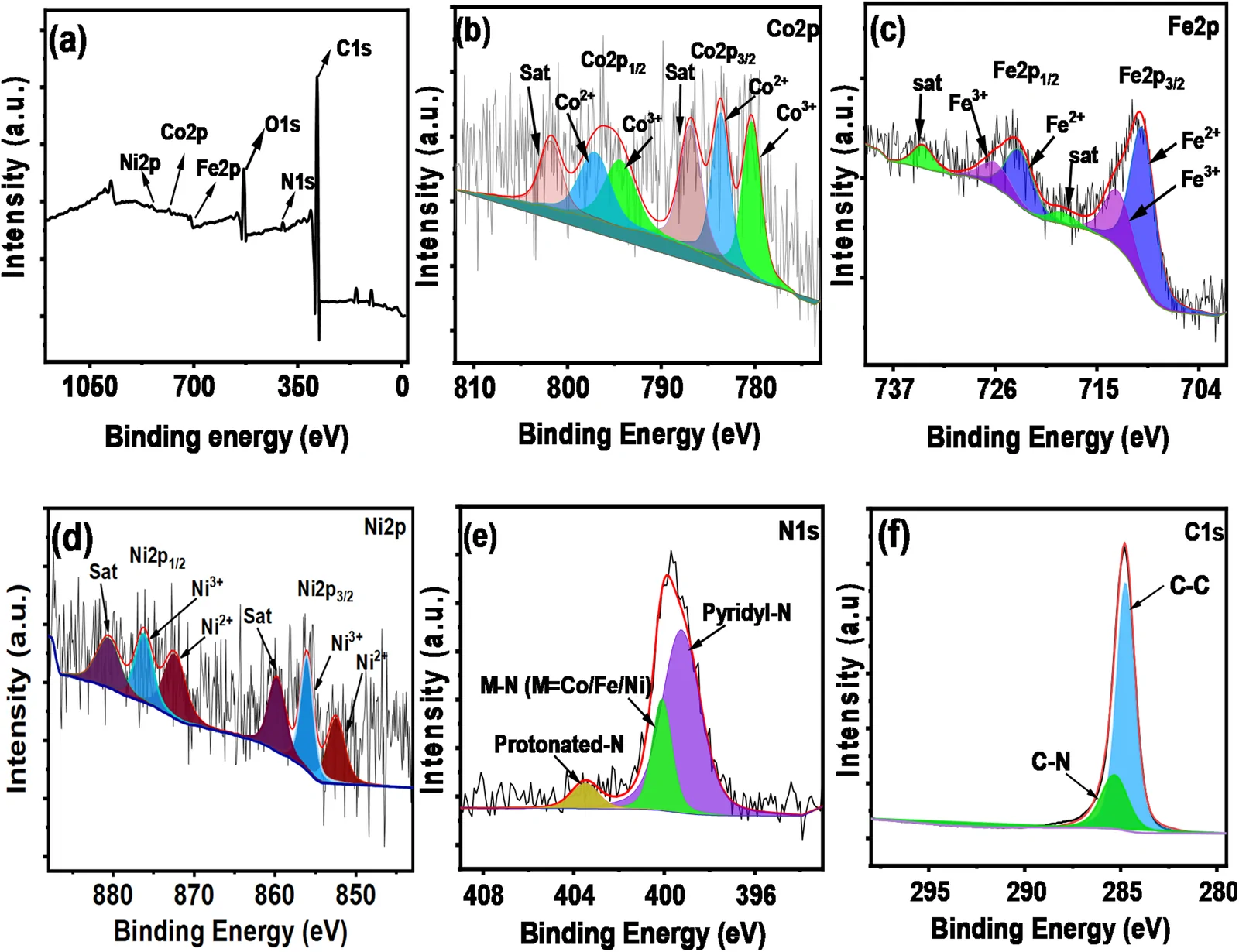
XPS study of polyCoFeNi (a) survey spectra that confirms the presence of three transition metals Co, Fe, Ni, along with O, N, and C elements. High-resolution spectra of (b) Co 2p (c) Fe 2p, (d) Ni 2p (e) N 1s (f) C 1s.

The Fe 2p spectrum (Fig. 3c) contains multiple components and satellite features characteristic of an Fe-containing coordination environment. The peaks at 710.2 and 713.1 eV are assigned to Fe^2+^ and Fe^3+^ species associated with the Fe 2p_3/2_ orbital, while the peaks at 723.6 and 726.0 eV correspond to the Fe^2+^ and Fe^3+^ components of the Fe 2p_1/2_ orbital.^45^ In addition, the satellite peaks at 718.6 and 733.9 eV are characteristic of iron-containing coordination compounds.^45^

Similarly, the high-resolution Ni 2p spectrum (Fig. 3d) confirms the presence of Ni-containing species; its complex multiplet/satellite envelope does not by itself establish discrete Ni^2+^/Ni^3+^ populations. The peaks at 856.0 and 876.3 eV are attributed to Ni^3+^, whereas those at 852.5 and 872.5 eV correspond to Ni^2+^ in the Ni 2p_3/2_ and Ni 2p_1/2_ spin–orbit components, respectively.^46^ The accompanying satellite peaks at 859.8 and 880.7 eV further support the presence of multiple nickel oxidation states.^47^

The high-resolution N 1s spectrum of polyCoFeNi was deconvoluted into three components centered at approximately 399.2, 400.0, and 403.0 eV (Fig. 3e). The component at 399.2 eV is assigned to pyridine-type (pyridyl) N associated with the terpyridine moieties, whereas the component at 400.0 eV is attributed to metal-coordinated pyridyl N (M–N, M = Co, Fe, and Ni).^47^ The shift toward higher binding energy upon metal coordination is consistent with redistribution of electron density at the pyridyl N donor atoms upon coordination. The weaker component at approximately 403.0 eV is tentatively attributed to a higher-binding-energy nitrogen environment, potentially arising from protonated or strongly electronically perturbed pyridyl N.^47^ These N 1s features are consistent with the involvement of terpyridyl nitrogen atoms in metal coordination within polyCoFeNi.

The C 1s spectrum (Fig. 3f) displays two characteristic peaks at 284.7 and 285.2 eV, which are assigned to C–C and C–N bonds, respectively, confirming the preservation of the conjugated terpyridine polymer chain.^48^ The O 1s peak observed at 532.5 eV is associated with adsorbed oxygen species or residual solvent molecules.

To further distinguish polyCoFeNi from a simple physical mixture of the corresponding monometallic polymers, comparative XPS measurements were performed using a physical mixture of independently prepared polyCo, polyFe, and polyNi (Fig. S1). Although both samples contain Co, Fe, Ni, N, C, and O, systematic differences are observed in their high-resolution spectra. All fitted Co 2p, Fe 2p, Ni 2p, N 1s, and C 1s components of the physical mixture occur at lower binding energies than the corresponding components of polyCoFeNi. The Co and Ni core-level components exhibit binding-energy differences of approximately 0.1–0.9 and 0.3–0.6 eV, respectively, whereas larger differences of 1.3–2.5 eV are observed for the Fe 2p components. Importantly, the pyridyl-N and metal-coordinated N (M–N) components shift from approximately 398.9 and 399.8 eV in the physical mixture to 399.2 and 400.0 eV, respectively, in polyCoFeNi. A detailed comparison of the binding energies and corresponding binding-energy differences for polyCoFeNi and the physical mixture is provided in Table S1. These systematic differences indicate distinct local metal–ligand electronic environments in polyCoFeNi compared with a simple physical combination of the three monometallic polymers and are consistent with redistribution of electron density upon simultaneous heterometallic coordination. Nevertheless, XPS does not resolve the precise sequence or spatial distribution of Co, Fe, and Ni within individual polymer chains.

Overall, XPS confirms the presence of Co, Fe, Ni, C, N, and O and supports metal–nitrogen coordination within the bis-terpyridine-containing material. The spectra do not establish discrete mixed-valence populations or a specific metal sequence, and these assignments are therefore not used as the basis for the electrochemical mechanism.

### 3.2. Electrochemical properties of polyCoFeNi

The electrochemical properties of the polyCoFeNi-modified electrode were first evaluated by CV using 5 mM K_3_[Fe(CN)_6_] in 1 M KCl at a scan rate of 50 mV s^−1^ (Fig. 4a). Compared with the bare GCE, the polyCoFeNi_GCE exhibited substantially higher anodic and cathodic peak currents, showing that modification of the GCE alters the ferricyanide interfacial response and increases the measured peak currents. For the polyCoFeNi_GCE, the anodic and cathodic peaks were observed at 0.12 V and 0.21 V, with peak currents of 88.12 and −97.0 µA, respectively. The peak current ratio (*I*_pa_/*I*_pc_) of 0.91 indicates a highly reversible redox process, suggesting efficient charge-transfer kinetics at the electrode/electrolyte interface. Moreover, the smaller peak-to-peak separation observed for the polyCoFeNi_GCE relative to the bare GCE is consistent with a more facile ferricyanide response at the modified interface.

**Fig. 4:**
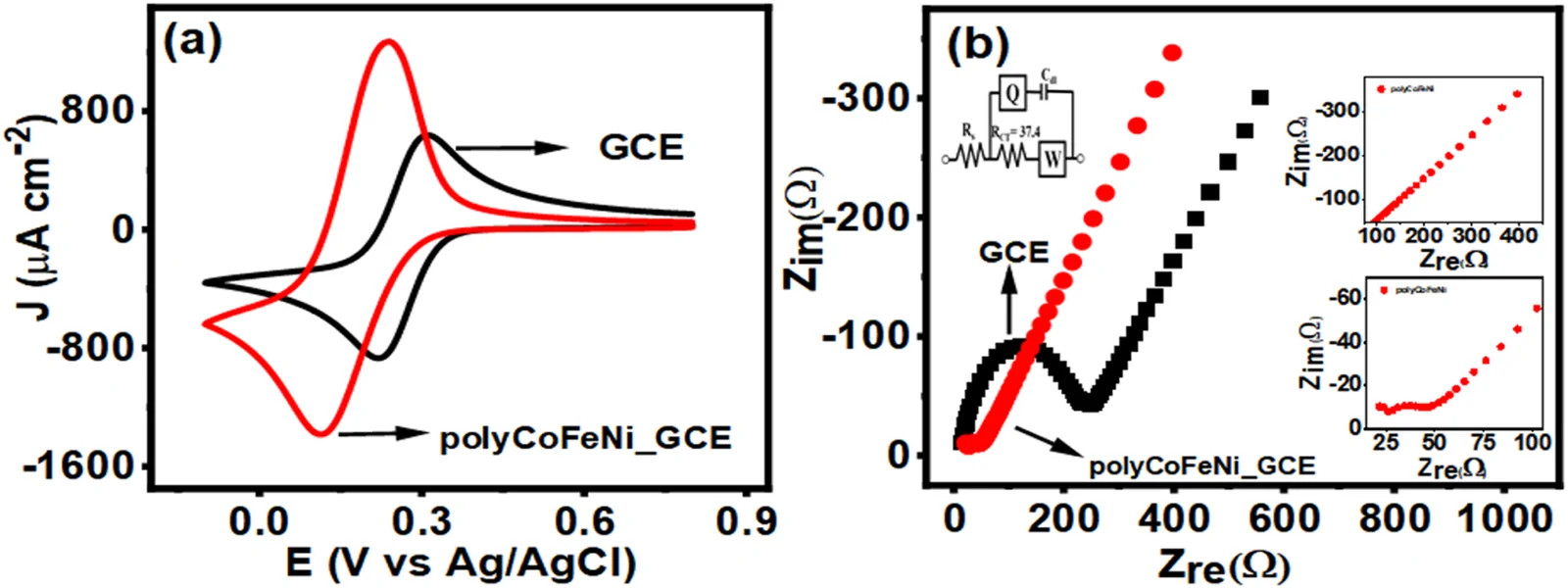
Electrochemical characterization of GCE and polyCoFeNi_GCE using (a) CV and (b) EIS in 5 mM K_3_[Fe(CN)_6_] containing 1 M KCl. The upper-right and lower-right insets show enlarged views of the low-frequency and high-frequency regions, respectively, of the polyCoFeNi_GCE Nyquist response.

To further evaluate the interfacial charge-transfer behavior, electrochemical impedance spectroscopy (EIS) was performed under the same electrolyte conditions (Fig. 4b). The Nyquist plot of the polyCoFeNi_GCE exhibits a much smaller semicircle in the high-frequency region than that of the bare GCE, indicating significantly lower charge-transfer resistance (*R*_ct_). The fitted *R*_ct_ values were 37.4 Ω for the polyCoFeNi_GCE and 209.4 Ω for the bare GCE. The lower fitted *R*_ct_ indicates that the modified surface provides a more favorable interface for the ferricyanide redox probe. Because ferricyanide kinetics can also be affected by surface roughness, adsorption, and film morphology, this result is not attributed uniquely to intrinsic redox activity of the metal centers. The data nevertheless support use of polyCoFeNi as an electrochemically accessible interface for subsequent 4-nitrophenol sensing.

### 3.3. Voltammetric response of 4-NP at bare and modified electrodes

The electrochemical response of 4-NP was first investigated by CV using the bare GCE and polyCoFeNi_GCE in 0.5 mM 4-NP solution at a scan rate of 50 mV s^−1^ over the potential range of 0.8 to −1.0 V (Fig. 5a and b). During the first forward scan, a pronounced cathodic peak (*R*_1_) appeared at approximately −0.77 V, corresponding to the irreversible reduction of the nitro group of 4-NP. Upon reversing the scan from −1.0 to 0.8 V, an anodic peak (O1) emerged at approximately 0.19 V, which is attributed to the oxidation of the electrochemically generated hydroxylaminophenol intermediate. In the subsequent cycle, a second cathodic peak (*R*_2_) was observed, arising from the reduction of the oxidized intermediate generated during the preceding anodic process. Consequently, the electrochemical behavior of 4-NP consists of an initial irreversible reduction (*R*_1_) followed by a reversible redox couple (O1/*R*_2_), which was observed at both the bare GCE and the polyCoFeNi_GCE.

**Fig. 5:**
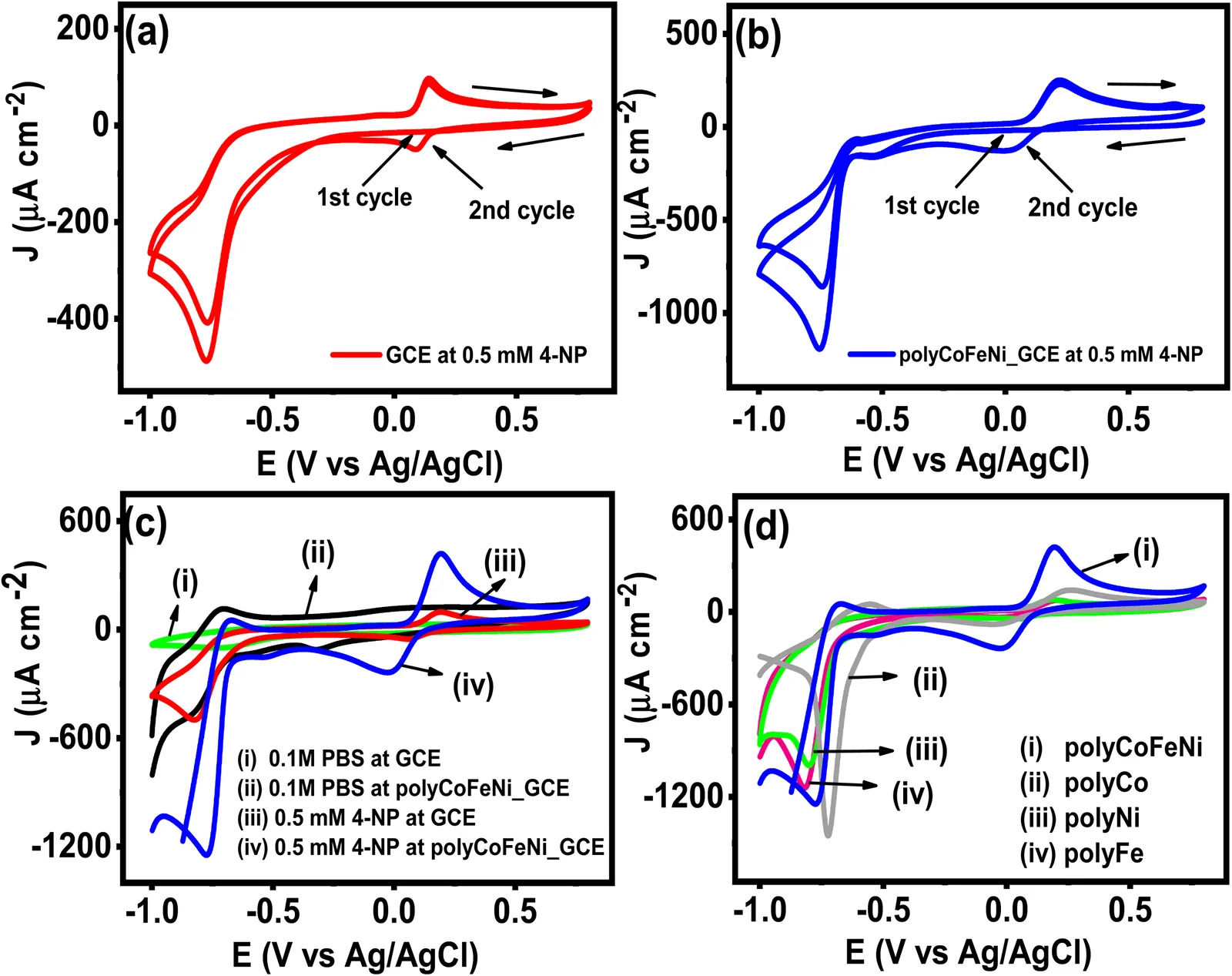
(a) CVs of GCE (a) and polyCoFeNi (b) in the 0.5 mM 4-NP at the scan rate of 50 mV s^−1^ for the first and second cycle. (c) Electrochemical performance of the bare GCE and polyCoFeNi_GCE in the absence and presence of 4-NP at the scan rate of 50 mV s^−1^. (d) CVs of 0.5 mM 4-NP in 0.1 M PBS at the polyFe_GCE, polyCo_GCE, and polyNi_GCE.

Compared with the bare GCE, the polyCoFeNi_GCE exhibited substantially higher peak current responses for all redox processes, showing that the polyCoFeNi-modified surface produces a larger electrochemical response toward 4-NP under the tested conditions. The enhanced response likely reflects a combination of the conjugated ligand, multiple metal-containing coordination environments, surface morphology, and analyte adsorption.The slight decrease in the *R*_1_ peak current during the second cycle is likely associated with partial adsorption of electrochemically generated intermediates on the electrode surface after the first reduction process.

To verify that the observed voltammetric responses originated exclusively from 4-NP, CV measurements were performed in 0.1 M PBS in the absence of 4-NP (Fig. 5c). No major redox peaks comparable in magnitude to the 4-NP signals were observed at either the bare GCE or the polyCoFeNi_GCE; weak background features of the modified electrode should nevertheless be considered when interpreting overlapping analytical peaks.

To further elucidate the role of each transition metal, the electrochemical responses of polyFe_GCE, polyCo_GCE, and polyNi_GCE were compared with that of polyCoFeNi_GCE under identical experimental conditions (Fig. 5d). The polyCoFeNi_GCE exhibited significantly higher anodic and reversible cathodic peak currents than the corresponding monometallic polymers, showing a higher O1/*R*_2_ response for the trimetallic material than for the investigated monometallic counterparts. Although the irreversible reduction peak (*R*_1_) recorded at the polyCo_GCE was slightly higher than that of the polyCoFeNi_GCE, its anodic response remained substantially lower. Because the O1 peak was reproducibly generated in every scan cycle and exhibited superior stability and analytical reproducibility, it was selected as the primary analytical signal for all subsequent electrochemical investigations unless otherwise stated. These comparisons show an improved O1/*R*_2_ response for the Co/Fe/Ni-containing material relative to the monometallic materials. Because bimetallic controls were not examined, the result is not taken as definitive evidence of a three-metal synergistic effect.

To further distinguish the electrochemical behavior of polyCoFeNi from that arising from a simple combination of the corresponding monometallic polymers, an additional control experiment was performed using a physical mixture of polyCo, polyFe, and polyNi (Fig. S1). The physical mixture exhibited a distinctly different voltammetric response from polyCoFeNi, including lower cathodic and anodic current responses and differences in the peak profiles and potentials. These observations indicate that the electrochemical behavior of polyCoFeNi is not reproduced by simple physical mixing of the three monometallic polymers and support the formation of a distinct Co/Fe/Ni-containing coordination environment.

### 3.4. pH variation and mass loading optimization

The influence of solution pH on the electrochemical response of 4-NP was investigated by CV in 0.5 mM 4-NP using 0.1 M PBS over the pH range of 3.0–9.0 at a scan rate of 50 mV s^−1^ (Fig. 6a). As shown in Fig. 6b, the anodic peak current (*J*_pa_) gradually increased from pH 3.0 to 5.0, indicating that mildly acidic conditions favor the electrochemical oxidation of 4-NP by promoting efficient proton-coupled electron-transfer reactions. Above pH 5.0, the peak current progressively decreased. This behavior is attributed to the gradual deprotonation of 4-NP at higher pH, which alters its adsorption characteristics and weakens its interaction with the electrode surface. In addition, the predominance of negatively charged 4-nitrophenolate species under alkaline conditions reduces the electrochemical response, resulting in lower oxidation currents.

**Fig. 6:**
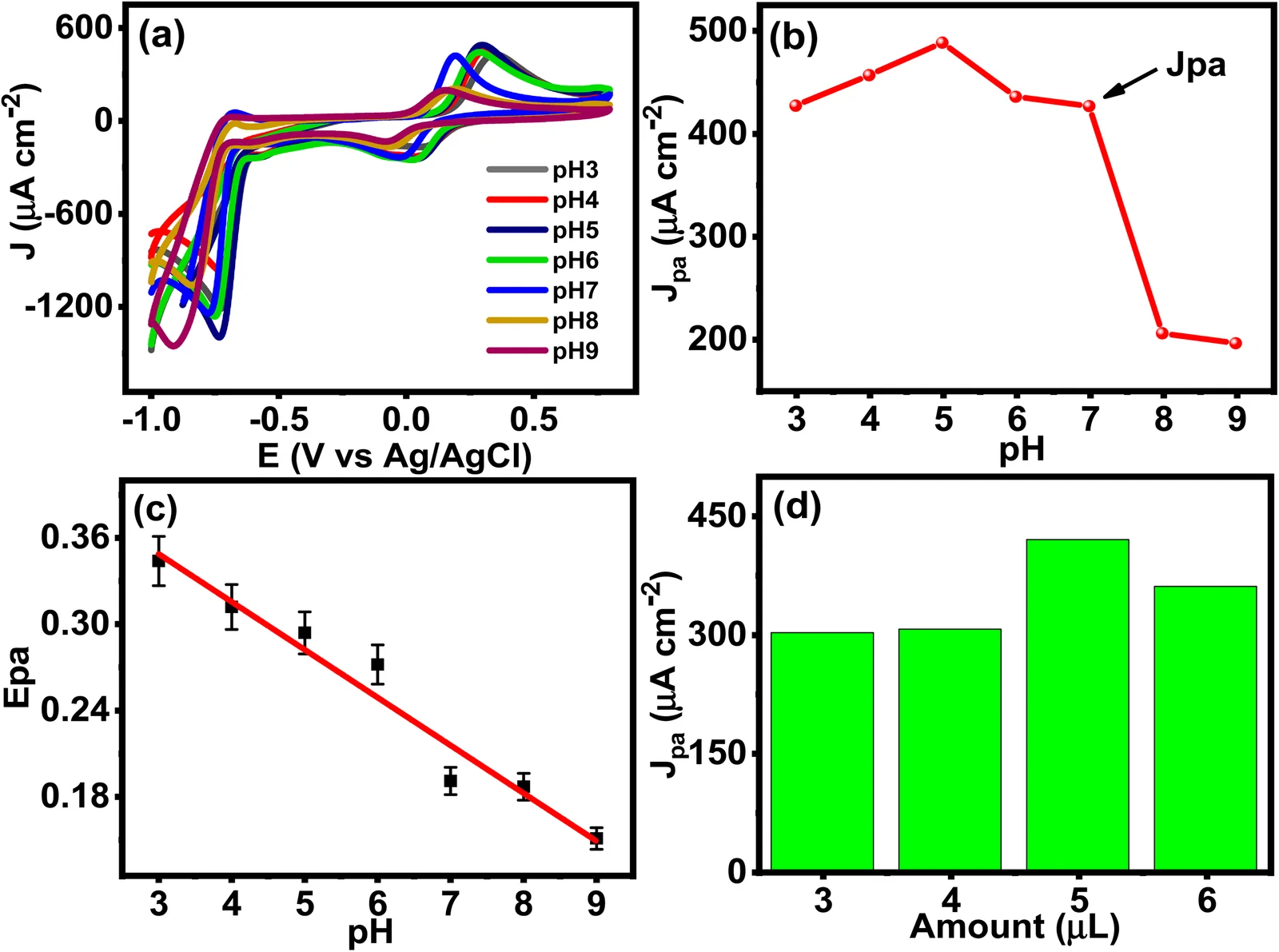
(a) CVs of 500 µM 4NP in polyCoFeNi_GCE at various pH levels ranging from pH 3 to pH 9. The plot of anodic peak current density (b) and peak potential (c) corresponding to pH. (d) Mass loading's impact on the peak current densities.

The dependence of the anodic peak potential on pH is presented in Fig. 6c, and is described by the following linear relationship:*E*_pa_ = −2.415 × pH + 43.286, *R*^2^ = 0.93

The negative shift in anodic peak potential with increasing pH confirms that proton transfer is involved in the oxidation process of 4-NP. A similar pH-dependent trend was also observed for the irreversible reduction peak (*R*_1_), indicating that both oxidation and reduction processes are proton-coupled. Although the highest anodic current was obtained at pH 5.0, pH 7.0 was selected as the optimum working condition because it provides a stable current response while offering greater practical relevance for environmental water analysis, where most real samples exist near neutral pH.

The effect of polyCoFeNi loading on the GCE was also investigated to optimize the electrochemical performance (Fig. 6d). As the deposited volume increased from 3.0 to 5.0 µL, the anodic peak current increased, indicating the progressive availability of more electroactive sites. However, further increasing the loading to 6.0 µL resulted in a decrease in current response. The excessive polymer layer likely increases film thickness, thereby impeding electron transport and slowing mass transfer across the electrode/electrolyte interface. Therefore, a loading of 5.0 µL was selected as the optimum amount for all subsequent electrochemical measurements.

### 3.5. Kinetics analysis of 4-NP

To elucidate the electron-transfer kinetics of the 4-NP redox process at the polyCoFeNi_GCE interface, CV was performed at scan rates ranging from 20 to 300 mV s^−1^ (Fig. 7a). As shown in Fig. 7b, the anodic and cathodic peak current lindensities increased early with the square root of the scan rate (*ν*^1/2^), following the relationships:*J*_pc1_ = −2.574 × 10^−4^ −7.314 × 10^−5^ × *v*^1/2^; *R*^2^ = 0.99*J*_pc2_ = 2.47 × 10^−6^ −1.912 × 10^−6^ × *v*^1/2^; *R*^2^ = 0.99*J*_pa_ = −6.290 × 10^−5^ + 5.484 × 10^−5^ × *v*^1/2^; *R*^2^ = 0.99

**Fig. 7:**
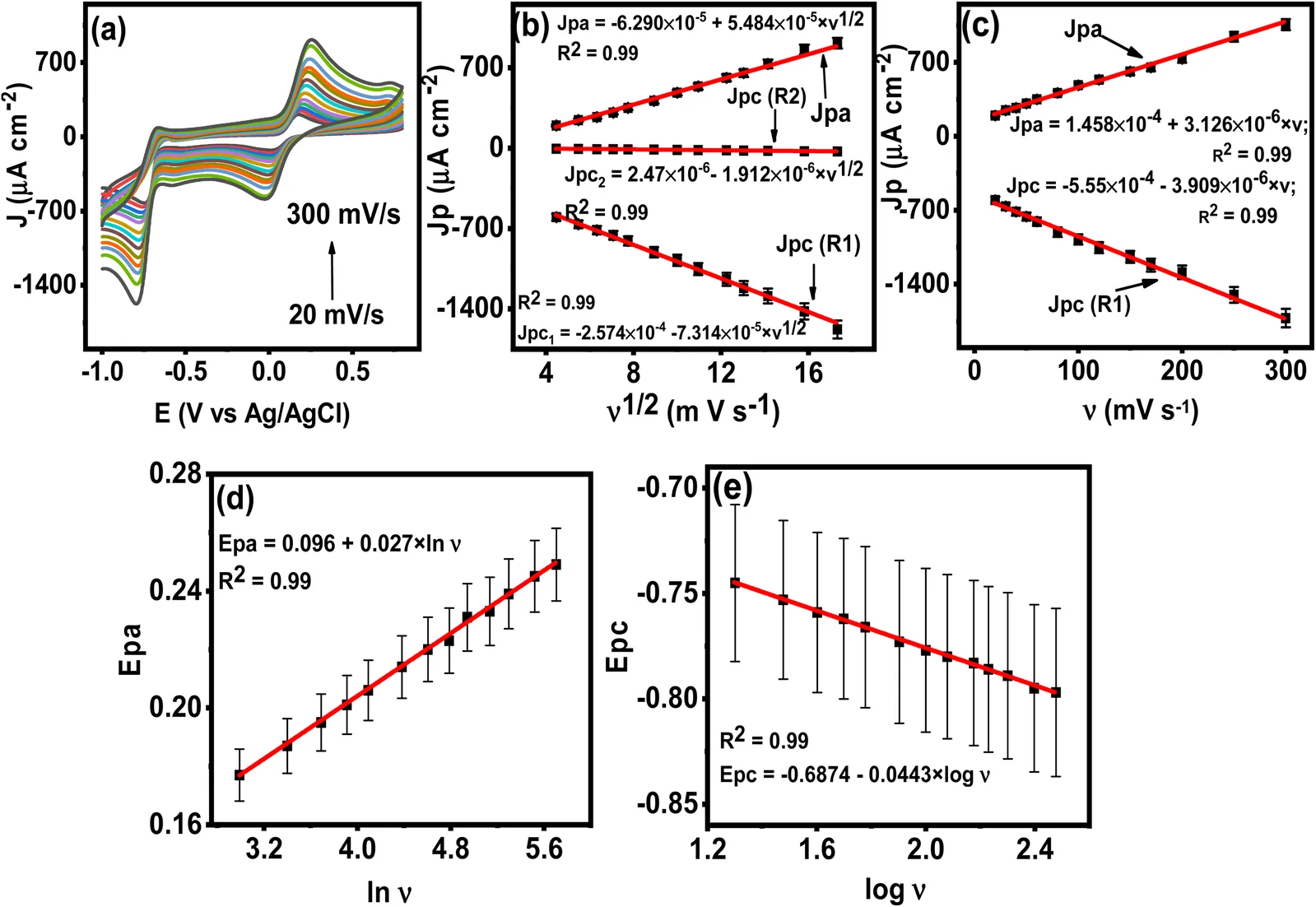
(a) The influence of scan rate on the CV responses regarding 0.5 mM 4-NP in PBS at the polyCoFeNi_GCE surface. The scan rates are 20, 30, 40, 50, 60, 80, 100, 120, 140, 170, 200, 250, and 300 mV s^−1^ (b) Peak current densities [*J*_pa_, *J*_pc_ (*R*_2_), and *J*_pc_ (*R*_1_)] *vs. ν*^1/2^. (c) Anodic and cathodic current *vs.* scan rate (d) anodic and (e) cathodic peak potential *vs.* ln *ν* and log *ν*.

The linear correlations with *ν*^1/2^ indicate a substantial diffusion contribution to the electrochemical response.^8^ However, the linear dependence of both anodic and cathodic peak currents on the scan rate (*ν*) (Fig. 7c) also suggests the contribution of an adsorption-controlled process. Therefore, the electrochemical oxidation and reduction of 4-NP at the polyCoFeNi_GCE proceed through a mixed diffusion–adsorption controlled mechanism.

An apparent electron number for the oxidation process was estimated using the Randles–Ševčík relationship as a diffusion-dominant approximation.2
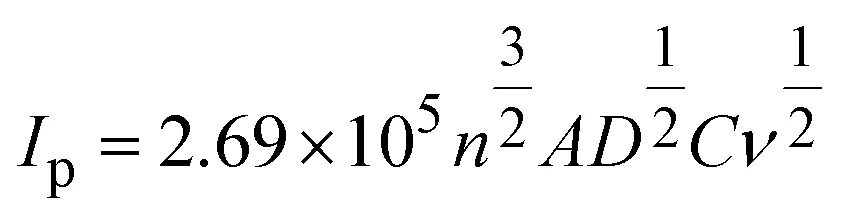
where *A*, *D*, *C*, *n*, and *ν* represent the electroactive surface area, diffusion coefficient, analyte concentration, number of transferred electrons, and scan rate, respectively. From the slope of the *J*_pa_*vs. ν*^1/2^ plot (Fig. 7b), an apparent *n* value of 1.7 was obtained, consistent with an approximately two-electron oxidation. Because the scan-rate analysis also indicates an adsorption contribution, this value is treated as supportive rather than definitive.

To further evaluate the electron-transfer kinetics, the charge-transfer coefficient (*α*) for the oxidation process was determined using the Laviron model (eqn (3)).^49^ The relationship between anodic peak potential and the natural logarithm of the scan rate (Fig. 7d) is expressed as:*E*_pa_ = 0.027 × ln *v* + 0.0956; *R*^2^ = 0.993
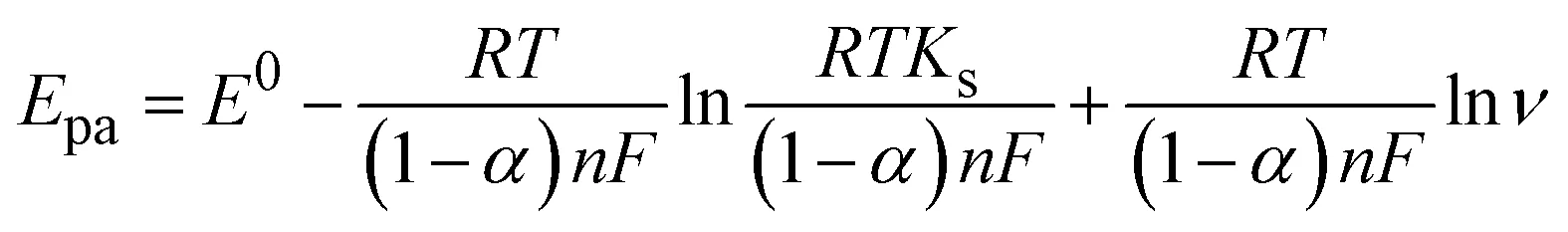
where *R*, *T*, *F*, *α*, *ν*, *K*_s_, *n*, and *F* have their usual meanings. The calculated *α* value was 0.52, and the apparent heterogeneous electron-transfer rate constant (*K*_s_), obtained from the Laviron treatment, was 13.8 × 10^2^ s^−1^. Because adsorption contributes to the overall response, these model-derived parameters are interpreted comparatively rather than as absolute intrinsic kinetic constants at the polyCoFeNi_GCE interface.

The reduction process was analyzed using the Laviron equation for an irreversible electrochemical system,^50^ and plotted *E*_p_*vs.* log *ν* (Fig. 7e), The dependence of cathodic peak potential on the logarithm of the scan rate (Fig. 7e) is given by:*E*_pc_ = −0.0443 × log *v* −0.6874; *R*^2^ = 0.994
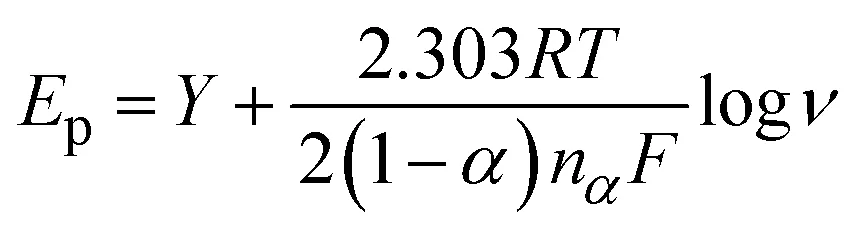


The calculated charge-transfer coefficient for the cathodic process was *α* = 0.34, consistent with an irreversible electron-transfer process under the applied model. Together, these analyses indicate that the polyCoFeNi-modified electrode supports kinetically accessible oxidation and reduction processes; the numerical kinetic parameters should be interpreted within the assumptions of the applied models.

### 3.6. Mechanistic pathway of the 4-NP redox process

A possible electrochemical pathway for 4-NP oxidation and reduction at the polyCoFeNi_GCE interface is proposed based on the observed peak sequence, pH dependence, scan-rate behavior, and established nitrophenol electrochemistry. The scheme is intended as a mechanistic interpretation rather than direct identification of every intermediate.

Upon application of the potential, 4-NP molecules diffuse from the bulk solution to the polyCoFeNi_GCE surface and become adsorbed through multiple non-covalent interactions. These interactions likely include π–π stacking between the aromatic ring of 4-NP and the conjugated bis-terpyridine ligand, possible interactions between the nitro/phenolic functionalities of 4-NP and polar metal–ligand sites at the modified surface, and hydrogen bonding between the hydroxyl group of 4-NP and surface oxygen-containing species or coordinated ligands. These non-covalent and interfacial interactions may contribute to adsorption of 4-NP and facilitate proton-coupled electron-transfer (PCET) processes.

During the first cathodic scan (0.8 → −1.0 V), an irreversible reduction peak (*R*_1_) appears at approximately −0.772 V, corresponding to the four-electron/four-proton reduction of 4-NP to 4-hydroxylaminophenol (4-HAP). Consistent with commonly reported nitrophenol electrochemistry, this overall transformation is proposed to proceed through a short-lived 4-nitrosophenol (4-NSP) intermediate. The assignment of the overall *R*_1_ process as a four-electron/four-proton reduction is therefore based on the established reaction pathway together with the observed electrochemical behavior, rather than on direct electron-count determination for *R*_1_ in the present study. Owing to its transient nature, 4-NSP is expected to undergo further reduction to 4-HAP. The overall reduction process can be represented as:OH-PhNO_2_ + 4e + 4H^+^ → OH-PhNHOH

During the reverse anodic scan (−1.0 → 0.8 V), a fraction of the accumulated 4-HAP is oxidized to 4-NSP, producing the anodic peak (O1) at approximately 0.19 V through a two-electron/two-proton process. In the subsequent cathodic scan, the generated 4-NSP is reduced back to 4-HAP, giving rise to the reversible cathodic peak (*R*_2_) at approximately −0.018 V (Scheme 3). The reversible redox transformation can be expressed as:OH-PhNHOH ⇌ OH-PhNO + 2H^+^ + 2e

**Scheme 3:**
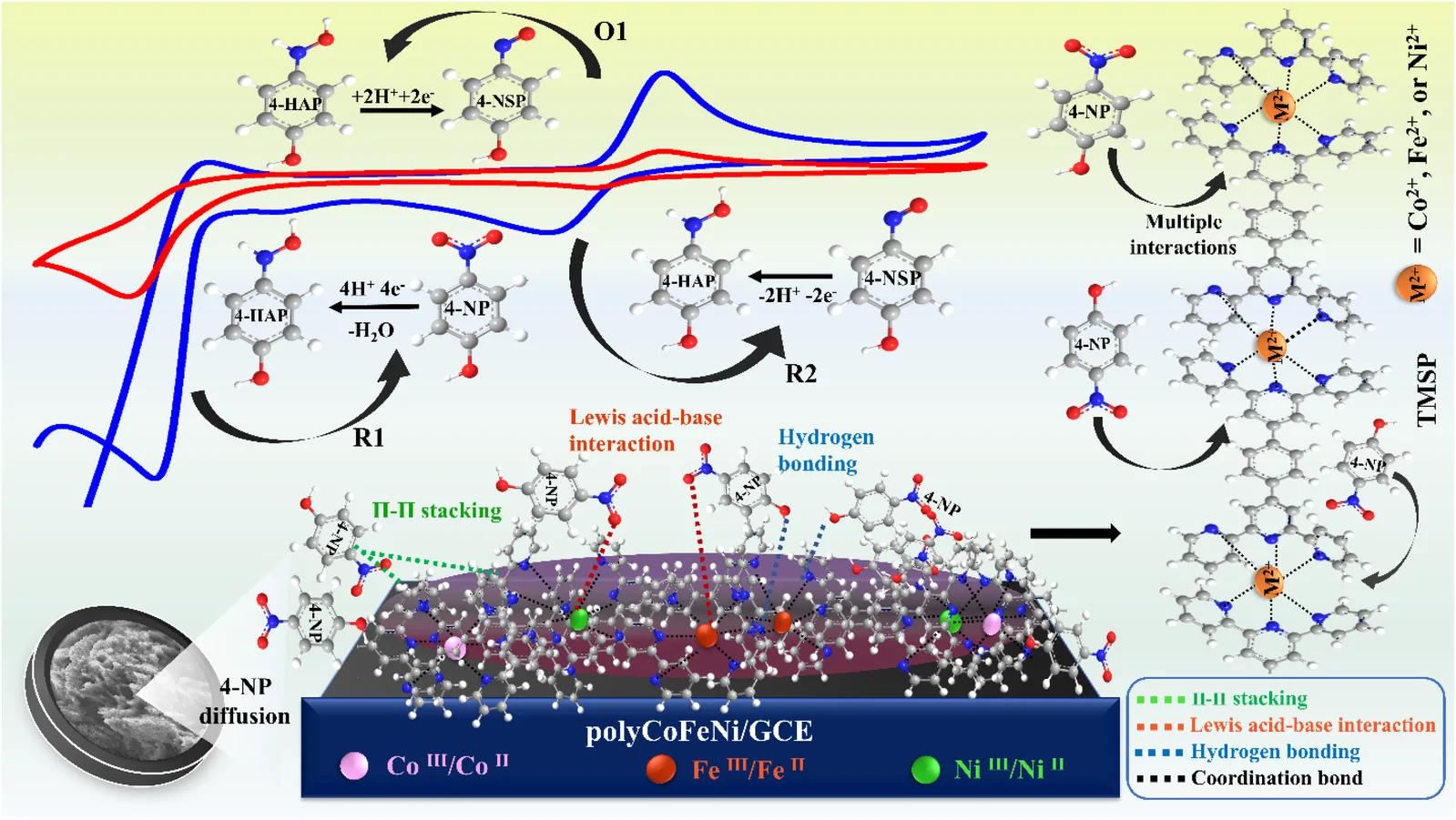
Proposed electrochemical pathway for 4-NP at polyCoFeNi_GCE interface.

The proposed pathway is consistent with the mixed diffusion–adsorption behavior and pH-dependent peak shifts. The conjugated bis-terpyridine polymer chain, multiple metal-containing coordination environments, and surface morphology may collectively influence analyte adsorption and interfacial charge transfer. The available data do not establish a unique metal-centered catalytic cycle or a three-metal synergistic mechanism. Accordingly, the available data support an enhanced O1/*R*_2_ response of polyCoFeNi relative to the investigated monometallic materials without assigning a unique three-metal catalytic mechanism.

### 3.7. Quantification of 4-NP

The quantitative sensing performance of the polyCoFeNi_GCE toward 4-NP was evaluated by cyclic voltammetry using 4-NP concentrations ranging from 2 to 2000 µM in 0.1 M PBS at a scan rate of 50 mV s^−1^. As shown in Fig. 8a, both the anodic and cathodic peak current densities increased progressively with increasing 4-NP concentration, showing a clear concentration-dependent response of the polyCoFeNi_GCE. The inset figures clearly illustrate the systematic increase in both oxidation and reduction peak currents. A wide linear response was obtained over the entire concentration range of 2–2000 µM, demonstrating the broad working range of the proposed sensing platform.

**Fig. 8:**
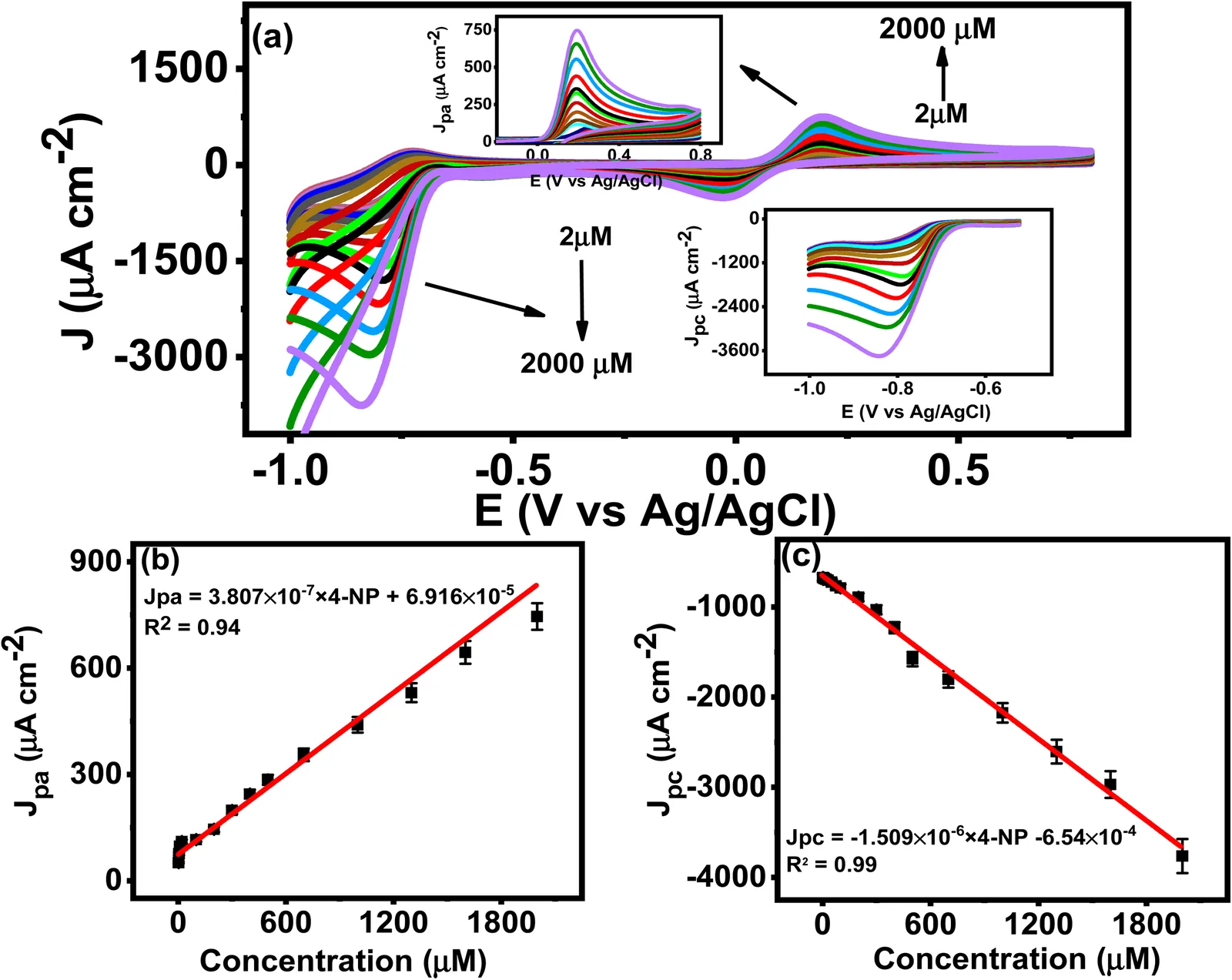
(a) The CV curves obtained for various concentrations (2, 5, 10, 30, 50, 75, 100, 200, 300, 500, 700, 1000, 1300, 1600, 2000 µM). The changes in anodic and cathodic peak current densities with 4-NP concentration are shown in the inset figures. The calibration plot of (b) anodic peak current density (*J*_pa_) and (c) cathodic peak current density (*J*_pc_) *vs.* concentration for the same concentration variation at a scan rate of 50 mV s^−1^.

The anodic calibration plot (Fig. 8b) exhibited a linear relationship between the anodic peak current density and 4-NP concentration, which can be expressed as:*J*_pa_ = 3.807 × 10^−7^[conc] + 6.916 × 10^−5^; *R*^2^ = 0.94

The limit of detection (LOD) was calculated using eqn (5):5
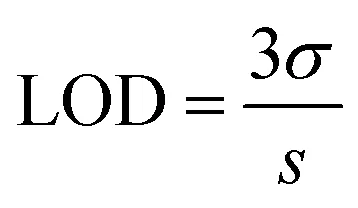
where *σ* and *s* represent the standard deviation of the blank response and the slope of the calibration plot, respectively. Based on this equation, the anodic process exhibited an LOD of 0.28 µM, indicating sensitive anodic detection of 4-NP with the polyCoFeNi_GCE.

Similarly, the cathodic calibration plot (Fig. 8c) also showed excellent linearity and is described by,*J*_pc_ = −1.509 × 10^−6^ × [conc] −6.54 × 10^−4^; *R*^2^ = 0.99

The corresponding LOD for the cathodic process was calculated to be 0.65 µM, showing that both oxidation and reduction signals can be used for quantitative determination of 4-NP.

To further assess the analytical performance of the proposed sensor, its sensing characteristics were compared with those of previously reported electrochemical platforms (Table 1). The polyCoFeNi_GCE provides a broad linear range (2–2000 µM) while maintaining a low detection limit (0.28 µM) using CV. Table 1 is intended to provide contextual comparison with representative reported sensors; differences in technique, electrode architecture, electrolyte, and calibration protocol should be considered when comparing analytical figures of merit directly.

**Table 1:** Comparison of the performance of polyCoFeNi_GCE with previously reported modifiers for 4-NP detection

Modified electrode	Method	Linear range (µM)	LOD (µM)	pH	References
PRB/PGE	CV, DPV	5.0–700.0	1.78	7.2	51
Pt NP-embedded PPy-CB@ZnO NCs	CV, DPV	1.5–40.5	1.25	7	52
PPy/AuNPs	CV, LSV	0.1 to 120	16.5	4	53
MIP	CV, amperometry	2–400	0.2		54
Cu0.5–Fe3O4@VXC-72/GCE	CV, DPV	0.1–4.0 and 5–150	0.065	6	55
Ni–CoOX NPs/GCE	DPV	7–682	4.8	6	56
CeO_2_ : Cu/GCE	CV, EIS	7.18–5000	7.18	7	57
polyCoFeNi	CV	2–2000	0.28	7	This work
			0.65		

PRB/PGE: poly-riboflavin modified pencil graphite electrode; Ppy-CB: polypyrrole-carbon black; MIP: molecular imprinted polymer.

### 3.8. Stability, reproducibility, and interference study

The operational stability of the polyCoFeNi_GCE was first evaluated by performing 40 consecutive CV cycles in 0.5 mM 4-NP at a scan rate of 50 mV s^−1^ (Fig. 9a). The voltammograms exhibited nearly overlapping anodic and cathodic peaks throughout the repeated measurements, indicating excellent electrochemical stability. The anodic peak current showed a relative standard deviation (RSD) of only 2.4%, indicating good operational stability for the oxidation of 4-NP. Similarly, the cathodic process exhibited a slightly higher but still low RSD of 4.8%, supporting good stability of the polyCoFeNi_GCE during the reduction process. The storage stability of the modified electrode was further examined after 7 days of storage (Fig. 9b). No significant change in the peak current response was observed, indicating that the polyCoFeNi_GCE retains its electrochemical activity over time.

**Fig. 9:**
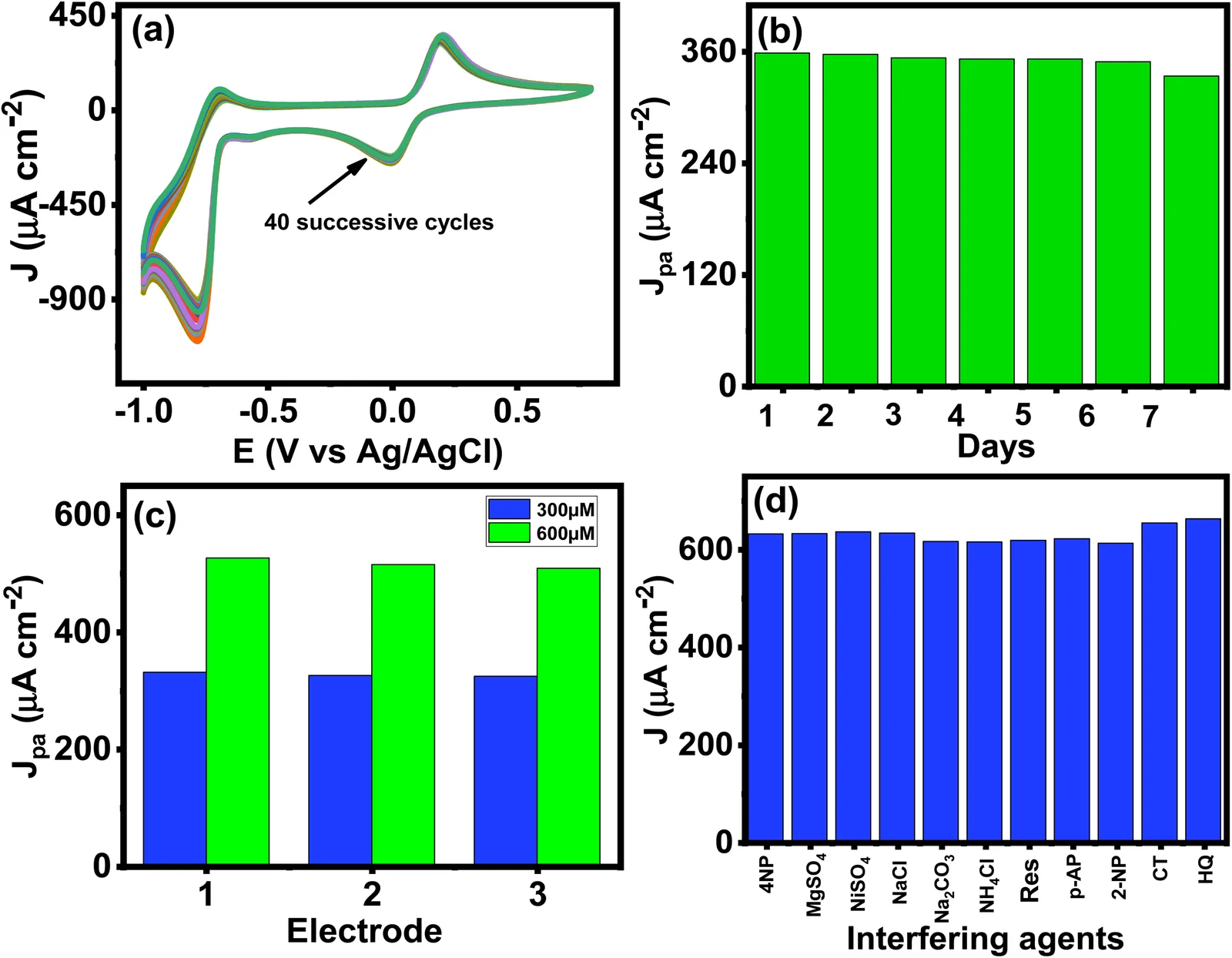
(a) Stability of polyCoFeNi through 40 successive CVs at 50 mV s^−1^. (b) Storage stability for 7 days. (c) Repeatability test of 4-NP detection with three different electrodes at varying concentrations (300 and 600 µM) (d) peak current density responses to 4-NP in the presence of various interfering agents, namely (i) Na_2_CO_3_, (ii) MgSO_4_, (iii) NaCl, (iv) NH_4_Cl, (v) NiSO_4_, (vi) *p*-AP, (vii) resorcinol, (viii) 2-NP (ix) catechol (CT) (x) hydroquinone (HQ).

The reproducibility of the proposed sensor was assessed using three independently prepared polyCoFeNi_GCEs in 300 and 600 µM 4-NP solutions under identical experimental conditions (Fig. 9c). The anodic current responses were highly consistent among the independently fabricated electrodes, yielding RSD values of 3.4% and 3.7% for 300 and 600 µM 4-NP, respectively. These low RSD values indicate good fabrication reproducibility of the proposed sensing platform.

To further evaluate the chemical stability of polyCoFeNi after the stability and reproducibility studies, post-stability XPS analysis was performed (Fig. S3), and the corresponding binding energies are compared in Table S2. The Co 2p and Fe 2p components exhibit essentially unchanged binding energies, while the pyridyl-N, metal-coordinated N (M–N), and C 1s components are also retained without appreciable shifts. In contrast, only minor binding-energy changes of approximately 0.2–0.3 eV are observed for the Ni 2p components, suggesting a slight perturbation of the local Ni electronic environment during electrochemical operation. Overall, the preservation of the principal XPS features indicates that the local metal–terpyridine coordination environment of polyCoFeNi is largely maintained after the stability and reproducibility studies.

The selectivity of the polyCoFeNi_GCE was evaluated in the presence of common inorganic salts and structurally related organic compounds, including MgSO_4_, NiSO_4_, NaCl, Na_2_CO_3_, NH_4_Cl, resorcinol, *p*-aminophenol, 2-nitrophenol (2-NP), 3-nitrophenol (3-NP), catechol, and hydroquinone. The electrochemical response was measured in the presence of each interfering species at tenfold higher concentration than that of 0.5 mM 4-NP. As shown in Fig. 9d, all interfering species produced relative errors below 5%, indicating good selectivity of the polyCoFeNi_GCE toward 4-NP. Slightly larger deviations were observed in the presence of resorcinol and 2-NP, which can reasonably be attributed to their structural similarity and competitive adsorption with 4-NP at the electrode surface. Nevertheless, the proposed sensor maintained high analytical accuracy in the presence of both inorganic ions and structurally related phenolic compounds, supporting its anti-interference capability under the tested conditions.

### 3.9. Real sample analysis

To evaluate the practical applicability of the proposed polyCoFeNi_GCE, real water samples collected from different sources, including tap water, lake water, and river water, were analyzed. The samples were spiked with 100 and 150 µM 4-NP and analyzed under the optimized experimental conditions using cyclic voltammetry. The concentrations of 4-NP were determined from the corresponding calibration curve, and the analytical recoveries were calculated. The results are summarized in Table 2.

**Table 2:** Determination of 4-NP from tap, lake, and river water

Samples	4-NP added (µM)	4-NP found (µM)	Recovery (%)	RSD (%)
Tap water	100	100.14	100.14%	0.66%
	150	149.91	99.94%	1.09%
Lake water	100	99.10	99.10%	1.03%
	150	151.21	100.80%	2.69%
River water	100	101.12	101.12%	2.33%
	150	148.72	99.14%	1.33%

The recoveries for the three water samples were generally close to 100%, with relative standard deviations (RSDs) below 3%, supporting matrix compatibility under the tested spike-recovery conditions. These measurements should be interpreted as spike-recovery tests rather than comprehensive environmental-method validation.

## 4. Conclusion

In this work, a novel bis-terpyridine-based trimetallic Co/Fe/Ni metallo-supramolecular polymer (polyCoFeNi) was synthesized and evaluated as an electrode modifier for electrochemical detection of 4-NP. UV-vis spectroscopy, FTIR, FESEM, EDS, XPS, and viscosity measurements support formation of a Co/Fe/Ni-containing bis-terpyridine supramolecular material with a rough, sponge-like surface morphology and metal–nitrogen coordination environments. The available characterization confirms the presence of all three metals but does not establish an ordered Co–Fe–Ni sequence or discrete mixed-valence populations; accordingly, these structural features are not overinterpreted.

The polyCoFeNi-modified electrode produced a larger O1/*R*_2_ response toward 4-NP than the bare GCE and the investigated monometallic analogues, with an initial irreversible reduction followed by a reversible oxidation/reduction couple. Scan-rate analysis indicated mixed diffusion–adsorption control. The improved response is likely associated with the combined influence of the conjugated bis-terpyridine polymer chain, multiple metal-containing coordination environments, surface morphology, and analyte adsorption; the present data are not taken as definitive evidence of a three-metal synergistic catalytic mechanism.

The resulting sensing platform demonstrated excellent analytical performance, providing a wide linear range of 2–2000 µM with low limits of detection of 0.28 µM and 0.65 µM for the anodic and cathodic processes, respectively, together with good stability, reproducibility, and interference tolerance. Furthermore, spike-recovery measurements in tap, lake, and river water demonstrate matrix compatibility of the polyCoFeNi-modified electrode for 4-NP determination. Overall, this study shows that Co/Fe/Ni-containing bis-terpyridine metallo-supramolecular materials can serve as useful electrode modifiers for electrochemical sensing when their structural and mechanistic claims are interpreted within the limits of the available characterization.

## Conflicts of interest

There are no conflicts of interest to declare.

## Supplementary Material

RA-016-D6RA08328E-s001

## Data Availability

The data that supports the findings of this study are available from the corresponding author upon reasonable request. Supplementary information file (SI): XPS characterization of a physical mixture of independently prepared polyCo, polyFe, and polyNi. Comparison of the CV responses of polyCoFeNi_GCE and a GCE modified with a physical mixture of polyCo, polyFe, and polyNi, XPS characterization of polyCoFeNi after the stability and reproducibility studies. Comparison of XPS binding energies of polyCoFeNi and the physical mixture of polyCo, polyFe, and polyNi. Comparison of the XPS binding energies of polyCoFeNi before and after the stability and reproducibility studies. See DOI: https://doi.org/10.1039/d6ra08328e.
