# Supplementary material for: A bis-terpyridine-based trimetallic Co/Fe/Ni metallo-supramolecular polymer for electrochemical detection of 4-nitrophenol: kinetic and mechanistic insights
Source: RSC Adv. 2026 Oct 15;16(47):63103–19. doi: 10.1039/d6ra08328e (PMC13670145; doi:10.1039/d6ra08328e)
Supplement: Supplementary file 1 [file RA-016-D6RA08328E-s001.pdf]

Supplementary Information (SI) for RSC Advances.
This journal is © The Royal Society of Chemistry

Supporting Information

A Bis-terpyridine-Based Trimetallic Co/Fe/Ni Metallo-Supramolecular Polymer for Electrochemical Detection of 4-Nitrophenol: Kinetic and Mechanistic Insights

Najia Tahsin Kumkum[a,1], Surekha Chaudhary[b,1], Md. Monjorul Islam[a], Sheikh Jakia Nur Oishee[a], Abdul Awal[a], Md. Delwar Hossain[a,*], Bong-Joong Kim[b,*], A. J. Saleh Ahammad[a,b*]

[a]*Department of Chemistry, Jagannath University, Dhaka 1100, Bangladesh.*

[b]*Department of Materials Science and Engineering, Gwangju Institute of Science and Technology (GIST), Gwangju, 61005 Korea.*

[1]These authors contributed equally to this work.

*Corresponding authors *E-mail*:

delwar@chem.jnu.ac.bd (MDH); kimbj@gist.ac.kr (BJK); ajsahammad@chem.jnu.ac.bd (AJSA)

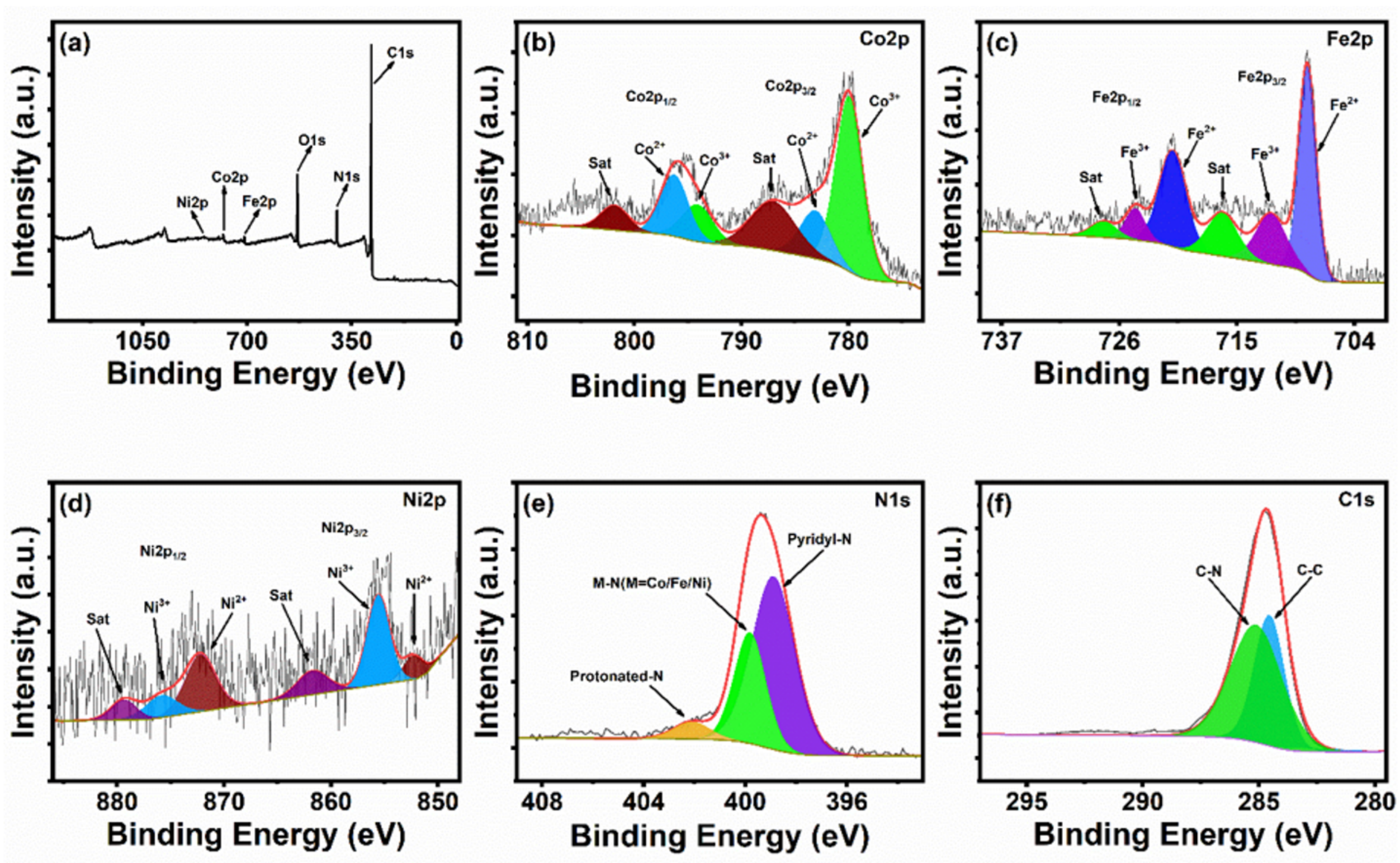

Figure S1. XPS characterization of a physical mixture of independently prepared polyCo, polyFe, and polyNi: (a) survey spectrum and high-resolution spectra of (b) Co 2p, (c) Fe 2p, (d) Ni 2p, (e) N 1s, and (f) C 1s.

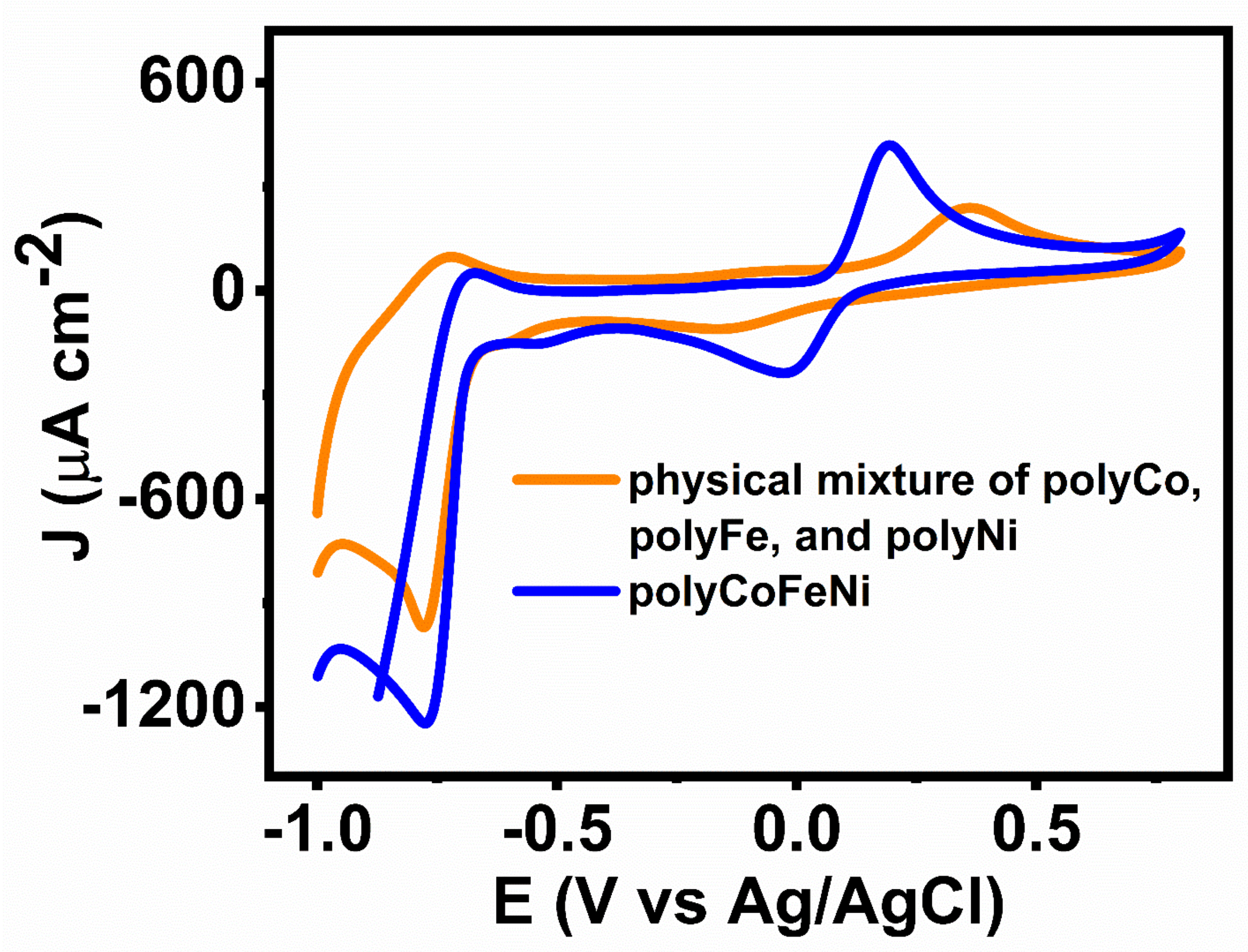

Figure S2. Comparison of the cyclic voltammetric responses of polyCoFeNi_GCE and a GCE modified with a physical mixture of polyCo, polyFe, and polyNi under identical experimental conditions in 0.5 mM 4-NP containing 0.1 M PBS at a scan rate of 50 mV s^{-1}.

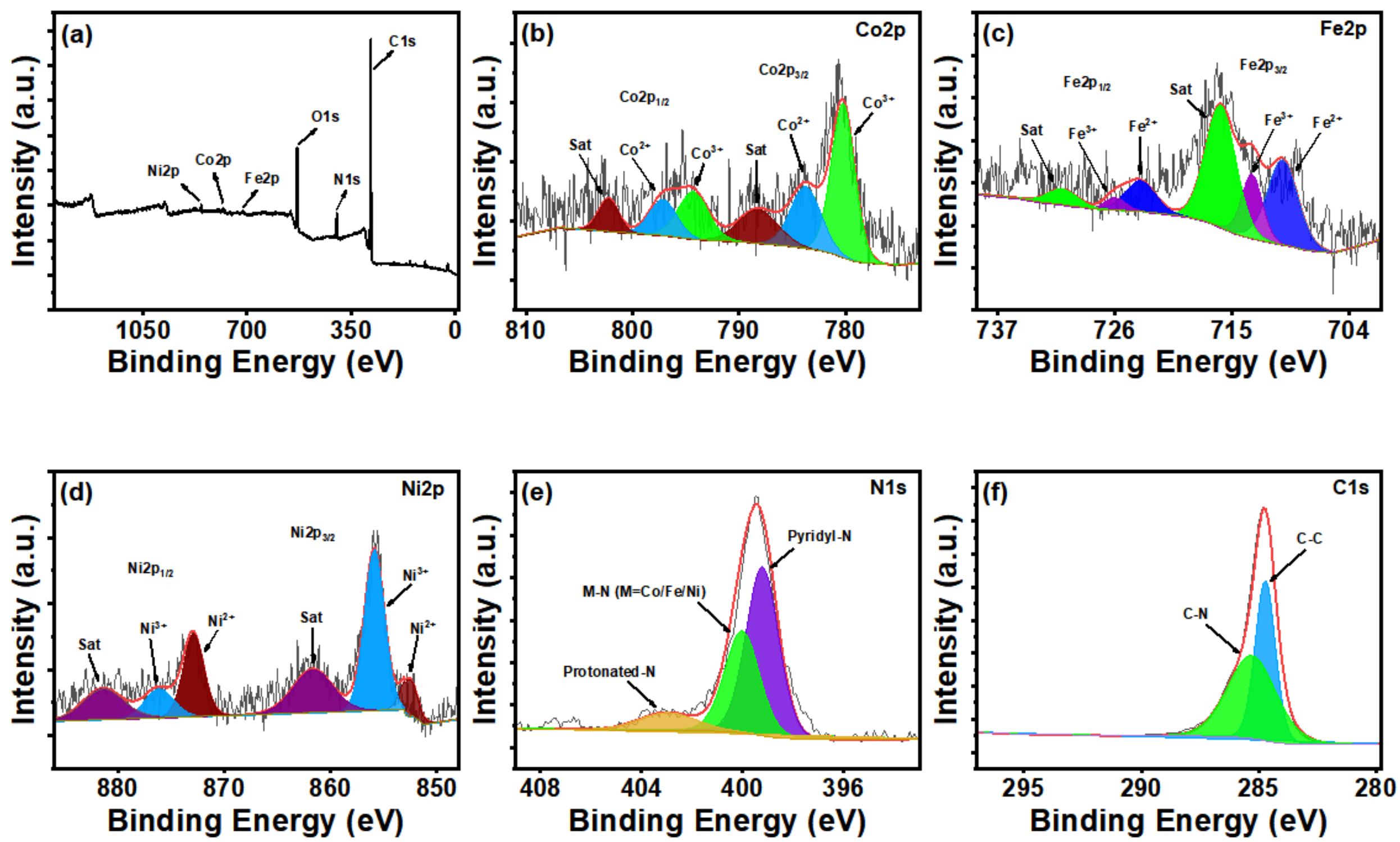

Figure S3. XPS characterization of polyCoFeNi after the stability and reproducibility studies: (a) survey spectrum and high-resolution spectra of (b) Co 2p, (c) Fe 2p, (d) Ni 2p, (e) N 1s, and (f) C 1s.

Table S1. Comparison of XPS binding energies of polyCoFeNi and the physical mixture of polyCo, polyFe, and polyNi.

Component	Binding energy of polyCoFeNi (eV)	Binding energy of physical mixture of polyCo, polyFe, and polyNi (eV)	Binding energy Shift (ΔBE) (eV)
$Co^{3⁺}$ 2p₃/₂	780.2	780	0.2
$Co^{2⁺}$ 2p₃/₂	783.7	783.1	0.6
$Co^{3⁺}$ 2p₁/₂	794.3	794.2	0.1
$Co^{2⁺}$ 2p₁/₂	797.1	796.2	0.9
$Fe^{3⁺}$ 2p₃/₂	713.1	711.8	1.3
$Fe^{2⁺}$ 2p₃/₂	710.2	708.4	1.8
$Fe^{3⁺}$ 2p₁/₂	726	724.4	1.6
$Fe^{2⁺}$ 2p₁/₂	723.6	721.1	2.5
$Ni^{3⁺}$ 2p₃/₂	856	855.5	0.5
$Ni^{2⁺}$ 2p₃/₂	852.5	852.2	0.3
$Ni^{3⁺}$ 2p₁/₂	876.3	875.7	0.6
$Ni^{2⁺}$ 2p₁/₂	872.5	872.1	0.4
N 1s (Pyridyl-N)	399.2	398.9	0.3
N 1s (M–N)	400	399.8	0.2
N 1s (Protonated-N)	403	402.1	0.9
C 1s (C–C)	284.7	284.5	0.2
C 1s (C–N)	285.2	285.1	0.1

Table S2. Comparison of the XPS binding energies of polyCoFeNi before and after the stability and reproducibility studies.

Component	Binding energy of polyCoFeNi (Before Stability) (eV)	Binding energy of polyCoFeNi (After Stability) (eV)	Binding energy Shift (ΔBE) (eV)
Co^{3}⁺ 2p₃/₂	780.2	780.2	0
Co^{2}⁺ 2p₃/₂	783.7	783.7	0
Co^{3}⁺ 2p₁/₂	794.3	794.3	0
Co^{2}⁺ 2p₁/₂	797.1	797.1	0
Fe^{3}⁺ 2p₃/₂	713.1	713.1	0
Fe^{2}⁺ 2p₃/₂	710.2	710.2	0
Fe^{3}⁺ 2p₁/₂	726	726	0
Fe^{2}⁺ 2p₁/₂	723.6	723.6	0
Ni^{3}⁺ 2p₃/₂	856	855.8	-0.2
Ni^{2}⁺ 2p₃/₂	852.5	852.7	0.2
Ni^{3}⁺ 2p₁/₂	876.3	876.1	-0.2
Ni^{2}⁺ 2p₁/₂	872.5	872.8	0.3
N 1s (Pyridyl-N)	399.2	399.2	0
N 1s (M–N)	400	400	0
N 1s (Protonated-N)	403	403	0
C 1s (C–C)	284.7	284.7	0
C 1s (C–N)	285.2	285.2	0
